# Functional characterization of 3β-hydroxysteroid dehydrogenases in Asiatic toad reveals an efficient broad substrate specificity of Bg-3βHSD2

**DOI:** 10.1042/BSR20260032

**Published:** 2026-08-07

**Authors:** Qian Meng, Yaoting Hu, Wenjie Chen, Chuanguang Xiao, Li Yang, Rufeng Wang, Beibei Zhang, Shujuan Zhao, Zhengtao Wang

**Affiliations:** State Key Laboratory of Discovery and Utilization of Functional Components in Traditional Chinese Medicine, The SATCM Key Laboratory for New Resources & Quality Evaluation of Chinese Medicine, The MOE Key Laboratory for Standardization of Chinese Medicines and Shanghai Key Laboratory of Compound Chinese Medicines, Institute of Chinese Materia Medica, Shanghai University of Traditional Chinese Medicine, Shanghai 201203, China

**Keywords:** 3β-hydroxysteroid dehydrogenase, amphibian steroid biosynthesis, bufadienolides, Bufo bufo gargarizans, enzyme functional diversity, steroid metabolism

## Abstract

3β-Hydroxysteroid dehydrogenases (3βHSDs) are key enzymes in steroid metabolism, catalyzing C3 oxidation–reduction and Δ^5^→Δ^4^ isomerization reactions that govern metabolic flux across multiple steroidogenic pathways. However, the functional diversity of 3βHSDs involved in bufadienolide metabolism in amphibians remains poorly explored. Here, we systematically characterized the *3βHSD* gene family in the Asian toad (*Bufo bufo gargarizans*) using integrated transcriptomic, biochemical, and metabolomic analyses. Seven *Bg-3βHSD* genes were identified from multi-tissue transcriptomes generated under control and Pb^2+^ exposure conditions, and six were heterologously expressed for functional evaluation. *In vitro* assays revealed pronounced functional divergence among Bg-3βHSD isoforms. Bg-3βHSD1 primarily catalyzed bidirectional C3 redox reactions of C21 steroids and bile acid-related substrates, consistent with canonical steroidogenic roles. In contrast, Bg-3βHSD2 enzyme exhibited broad substrate specificity and high catalytic efficiency toward hormones, bile acids, and bufadienolides. In addition to canonical C3 redox reactions and Δ^5^→Δ^4^ isomerization, Bg-3βHSD2 also displayed additional oxidation activity at the C17 position for several steroid substrates. A third homolog, Bg-HSD3B7 (GenBank accession no. XM 044303756.1), selectively converted 7α-hydroxylated sterols, suggesting a potential role in classical bile acid metabolism. Integration of tissue-specific expression profiles with bufadienolide distribution patterns suggests that Bg-3βHSD2 may contribute to connecting classical steroid metabolism with bufadienolide biosynthesis in adrenal tissue. Together, the present study identifies Bg-3βHSD2 as an efficient and versatile steroid-transforming enzyme, expands our knowledge of functional diversity within the amphibian 3βHSD family, and provides insights into the enzymatic basis of steroid and bufadienolide metabolism in *B. bufo gargarizans*.

## Introduction

3β-Hydroxysteroid dehydrogenases (3βHSDs; EC 1.1.1.145) are key enzymes in steroid metabolism, catalyzing the oxidation-reduction of the 3β-hydroxyl group and the Δ^5^→Δ^4^ isomerization of steroid substrates [1]. Through these reactions, 3βHSDs play central roles in multiple steroidogenic pathways, including the biosynthesis of glucocorticoids, mineralocorticoids, hormones, bile acids, and other steroid-derived metabolites [1–7]. In vertebrates, particularly mammals, the biochemical properties, tissue distribution, and physiological roles of 3βHSDs have been extensively investigated, establishing these enzymes as indispensable components of steroid homeostasis [5,8,9].

Accumulating evidence indicates that 3βHSDs exhibit considerable functional diversity across taxa. In addition to the well-characterized mammalian enzymes, 3βHSD homologs have been identified in a wide range of non-mammalian vertebrates, invertebrates, and microorganisms [10–14]. Comparative studies have revealed variations in substrate preference, cofactor utilization, and catalytic efficiency among different 3βHSDs [4,12,13], suggesting that these enzymes may have evolved to serve distinct metabolic requirements. Such diversity highlights the importance of examining 3βHSD function beyond classical model systems in order to gain a broader understanding of steroid metabolic flexibility.

Steroid metabolism remains largely unexplored in amphibians, despite their unique steroid profiles and physiological adaptations. The Asiatic toad (*Bufo bufo gargarizans*) is particularly notable for producing bufadienolides, a class of cardiotonic steroids characterized by diverse structural modifications [15]. These compounds are known to accumulate in multiple tissues and are believed to play important roles in chemical defense [16,17]. Previous studies have suggested that bufadienolide biosynthesis is closely related to bile acid and steroid hormone metabolism, involving cytochrome P450 (cytochrome P450 monooxygenase) enzymes and short-chain dehydrogenase/reductase (SDR) family members that catalyze redox-driven modifications of the steroid scaffold [15]. In particular, the conversion of C21 or C27 precursors into the characteristic C24 bufadienolide skeleton requires multiple sequential oxidation-reduction reactions [18–20]. 3βHSDs are members of the SDR superfamily and play a central role in steroid transformations, making them plausible candidates for involvement in bufadienolide biosynthesis [1,4–7]. Nevertheless, information on amphibian 3βHSDs is limited, and whether amphibian 3βHSDs possess substrate specificities and catalytic properties distinct from those of their mammalian counterparts remains unclear.

In *B. bufo gargarizans*, multiple putative *3βHSD* genes have been identified through genomic analyses. Among them, one *3βHSD* has been functionally characterized. This enzyme catalyzes the conversion of pregnenolone to progesterone and dehydroepiandrosterone to androstenedione but shows no detectable activity toward cholesterol or other sterols [20]. These findings suggest potential functional diversity within the 3βHSD family; however, these enzymes still lack systematic biochemical characterization. In particular, the respective roles of individual 3βHSD isoforms in steroid metabolism and their substrate selectivity toward bufadienolide-related intermediates remain largely unknown.

A significant challenge in understanding toad steroidogenesis is the existence of multiple enzyme paralogs that may have undergone functional divergence. In mammalian systems, 3βHSD isoforms exhibit tissue-specific expression and substrate preferences [3], but whether the expansion of the 3βHSD gene family in toads correlates with the complexity of bufadienolide metabolism is unknown. Furthermore, toads frequently encounter environmental stressors, such as heavy metal pollution, which can perturb their endocrine and metabolic flux and consequently alter the levels of defensive metabolites, including bufadienolides [21–23]. Investigating the transcriptional and metabolic response of the 3βHSD network under such stress is crucial for deciphering the regulatory mechanisms of bufadienolide homeostasis.

In the present study, we identified seven *3βHSD* genes (*Bg-3βHSD1, Bg-3βHSD2, Bg-3βHSD3, Bg-3βHSD4, Bg-3βHSD5, Bg-3βHSD6*, and *Bg-3βHSD7*) from *B. bufo gargarizans*. By integrating multi-tissue transcriptomics with targeted metabolomics, we uncovered distinct expression patterns and potential physiological roles of these isoforms. Functional analyses revealed marked differences in substrate specificity among Bg-3βHSD1, Bg-3βHSD2, and Bg-HSD3B7. Notably, Bg-3βHSD2 exhibited broad substrate promiscuity and a previously undescribed C17-oxidation activity, catalyzing the direct conversion of pregnenolone to dehydroepiandrosterone. This unexpected catalytic capability highlights the remarkable functional plasticity of amphibian 3βHSD enzymes and provides new insights into the diversification of steroidogenic pathways during vertebrate evolution.

## Materials and methods

### Reagents and chemicals

All steroid compounds (Table 1) used in the present study were purchased from the companies listed in Supplementary Table S1. The sources of the nucleotides NAD^+^ (nicotinamide adenine dinucleotide (oxidized form)), NADH (nicotinamide adenine dinucleotide (reduced form)), NADP^+^ (nicotinamide adenine dinucleotide phosphate (oxidized form)), and NADPH (nicotinamide adenine dinucleotide phosphate (reduced form)) are listed in Supplementary Table S2. The PCR amplification reagents and reverse transcription kit were purchased from Takara (China, Dalian).

**Table 1:** Steroid compounds and their chemical structures used in the present study

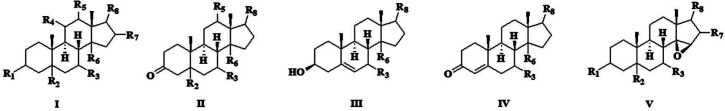										
Compounds	Name	Type	R_1_	R_2_	R_3_	R_4_	R_5_	R_6_	R_7_	R_8_
Hormones	Epiandrosterone (**1**)	I	β-OH	α-H	H	H	H	α-H	H	O
	Androstanedione (**2**)	II		α-H			H	α-H		O
	3β-Androstanediol (**3**)	I	β-OH	α-H	H	H	H	α-H	H	β-OH
	Androstanolone (**4**)	II		α-H			H	α-H		β-OH
	Epipregnanolone (**5**)	I	β-OH	β-H	H	H	H	α-H	H	β-COCH_3_
	5β-Pregnane-3,20-dione (**6**)	II		β-H			H	α-H		β-COCH_3_
	Androstenediol (**7**)	III			H			α-H		β-OH
	Testosterone (**8**)	IV			H			α-H		β-OH
	Dehydroepiandrosterone (**9**)	III			H			α-H		O
	Androstenedione (**10**)	IV			H			α-H		O
	Pregnenolone (**11**)	III			H			α-H		β-COCH_3_
	Progesterone (**12**)	IV			H			α-H		β-COCH_3_
	Allopregnanolone (**13**)	I	β-OH	α-H	H	H	H	α-H	H	β-COCH_3_
Oxysterols	Cholesterol (**14**)	III			H			α-H		β-CH_3_CH(CH_2_)_3_CH(CH_3_)_2_
	4-Cholesten-3-One (**15**)	IV			H			α-H		β-CH_3_CH(CH_2_)_3_CH(CH_3_)_2_
	7α-Hydroxycholesterol (**16**)	III			α-OH			α-H		β-CH_3_CH(CH_2_)_3_CH(CH_3_)_2_
	7α-Hydroxy-4-Cholesten-3-One (**17**)	IV			α-OH			α-H		β-CH_3_CH(CH_2_)_3_CH(CH_3_)_2_
	β-Sitosterol (**18**)	III			H			α-H		β-CH_3_CH(CH_2_)_2_CH(CH_2_CH_3_)CH(CH_3_)_2_
	Stigmasterin (**19**)	III			H			α-H		β-CH_3_CHCH = CHCH(CH_2_CH_3_)CH(CH_3_)_2_
	7β-Hydroxycholesterol (**20**)	III			β-OH			α-H		β-CH_3_CH(CH_2_)_3_CH(CH_3_)_2_
	27-Hydroxycholesterol (**21**)	III			H			α-H		β-CH_3_CH(CH_2_)_3_CH(CH_3_)CH_2_OH
	7α,27-Dihydroxycholesterol (**22**)	III			α-OH			α-H		β-CH_3_CH(CH_2_)_3_CH(CH_3_)CH_2_OH
Bile acids	Isolithocholic acid (**23**)	I	β-OH	β-H	H	H	H	α-H	H	β-CH_3_CH(CH_2_)_2_COOH
	Dehydrolithocholic acid (**24**)	II		β-H			H	α-H		β-CH_3_CH(CH_2_)_2_COOH
	Chenodeoxycholic acid (**25**)	I	α-OH	β-H	α-OH	H	H	α-H	H	β-CH_3_CH(CH_2_)_2_COOH
	Ursodeoxycholic acid (**26**)	I	α-OH	β-H	β-OH	H	H	α-H	H	β-CH_3_CH(CH_2_)_2_COOH
	Lithocholic acid(**27**)	I	α-OH	β-H	H	H	H	α-H	H	β-CH_3_CH(CH_2_)_2_COOH
	Cholic acid (**28**)	I	α-OH	β-H	β-OH	H	α-OH	α-H	H	β-CH_3_CH(CH_2_)_2_COOH
	7,12-Dihydroxy-3-oxocholanic acid (**29**)	II		β-H	α-OH		α-OH	α-H		β-CH_3_CH(CH_2_)_2_COOH
	Desoxycholic acid (**30**)	I	α-OH	β-H	H	H	α-OH	α-H	H	β-CH_3_CH(CH_2_)_2_COOH
Bufadienolide	Bufalin (**31**)	I	β-OH	β-H	H	H	H	β-OH	H	β-2H-pyran-2-one
	Bufotaline (**32**)	I	β-OH	β-H	H	H	H	β-OH	β-OAc	β-2H-pyran-2-one
	Arenobufagin (**33**)	I	β-OH	β-H	H	α-OH	O	β-OH	H	β-2H-pyran-2-one
	Gamabufotalin (**34**)	I	β-OH	β-H	H	α-OH	H	β-OH	H	β-2H-pyran-2-one
	Telocinobufagin (**35**)	I	β-OH	β-OH	H	α-OH	H	β-OH	H	β-2H-pyran-2-one
	Resibufogenin (**36**)	V	β-OH	β-H	H			β-OH	H	β-2H-pyran-2-one
	Cinobufagin (**37**)	V	β-OH	β-H	H			β-OH	β-OAc	β-2H-pyran-2-one
	Desacetylcinobufagin (**38**)	V	β-OH	β-H	H			β-OH	β-OH	β-2H-pyran-2-one
	Desacetylcinobufotalin (**39**)	V	β-OH	β-OH	H			β-OH	β-OH	β-2H-pyran-2-one
	Cinobufotalin (**40**)	V	β-OH	β-OH	H			β-OH	β-OAc	β-2H-pyran-2-one

### Animals and experimental procedures

Chinese toads (*B. bufo gargarizans* Cantor) were obtained from Jining, Shandong Province, China. All animal experiments were conducted at Shanghai University of Traditional Chinese Medicine. Toads were acclimatized for 7 days under controlled conditions (18°C–22°C, 60%–70% relative humidity, natural light cycle) and fed live mealworms daily.

After acclimation, six adult toads with similar body weights were randomly assigned to a control group (Bg) and PbSO_4_-exposed group (Cg) (*n* = 3 per group). The Bg group was maintained in tap water, whereas the Cg group was exposed to 5.46 mg/l PbSO_4_ in static water for 3 weeks, with complete water replacement every 3 days. At the end of exposure, toads were killed by intraperitoneal overdose of sodium pentobarbital (200 mg/kg body weight). Death was confirmed by sustained loss of corneal and righting reflexes and absence of spontaneous respiration. Parotoid gland, skin, liver, and adrenal tissues were collected after confirmed euthanasia, immediately snap-frozen in liquid nitrogen, and stored at −80°C until further analysis.

### RNA extraction and sequencing

Total RNA was extracted from parotoid gland, skin, liver, and adrenal tissues of toads using the TRIzol® kit (Invitrogen, U.S.A.). Samples from the control and PbSO_4_-exposed groups were designated as Bg_ear and Cg_ear (parotoid gland), Bg_skin and Cg_skin (skin), Bg_liver and Cg_liver (liver), and Bg_adr and Cg_adr (adrenal), respectively. RNA concentration and purity were assessed using a NanoDrop 2000 spectrophotometer (Thermo Scientific, U.S.A.), and RNA integrity was evaluated with an Agilent 2100 Bioanalyzer. Libraries were constructed following standard protocols, and paired-end sequencing was performed on an Illumina HiSeq 4000 platform.

### Transcriptome assembly and bioinformatic analysis

Raw sequencing reads were filtered to remove adapter sequences and low-quality reads using SeqPrep and Sickle (v1.2). Quality metrics, including base composition and error rates, were evaluated using the Fastx toolkit (v0.0.14). Clean reads were *de novo* assembled into transcripts using Trinity (v2.5.0), and assembly completeness was assessed with BUSCO. Gene expression levels were estimated by mapping clean reads back to the assembled transcripts.

Functional annotation of unigenes was performed against the Nr, Swiss-Prot, Pfam, COG, GO, and KEGG databases. Candidate βHSD family genes were identified based on functional annotations using the keywords ‘3βHSD’ and ‘HSD’ and validated by BLAST searches against the NCBI database. Physicochemical properties of the predicted proteins, including molecular weight, theoretical isoelectric point (pI), grand average of hydropathicity, and instability index, were calculated using the ExPASy ProtParam tool. The instability index was used to predict the *in vitro* stability of the proteins. Transmembrane domains were predicted using TMHMM, and subcellular localization was inferred using WoLF PSORT.

Amino acid sequences of functionally characterized 3βHSD proteins from other species were retrieved from UniProt. Phylogenetic analysis was performed using the maximum likelihood (ML) method in MEGA X with 1000 bootstrap replicates. Conserved motifs were identified using MEME (maximum number of motifs = 15; default parameters), and phylogenetic trees and motif distributions were visualized using TBtools.

### Transcriptional profile analysis of *Bg-3βHSD*

The expression profiles of the annotated *Bg-3βHSD*s transcripts across tissue and upon Pb^2+^ exposure were analyzed using transcriptomic data from the Pb^2+^-exposed group and the control group samples of *B. bufo gargarizans*. The transcriptomic data of different tissues in the control group were used for a gene tissue-specific expression assay. Differential expression analysis was performed using DESeq2 with an adjusted *P*-value (*P*adj) <0.05 and |log_2_FC| ≥1 as the threshold for significance.

### Plasmid and recombinant strain construction

Candidate *Bg-3βHSD1-5, Bg-3βHSD7*, and *Bg-HSD3B7* genes were cloned via PCR amplification using cDNA of Asiatic toad as templates and primers listed in Supplementary Table S3. The coding regions of these genes were separately ligated into pCold vector using T4 DNA ligase (Takara, Japan), yielding pCold-Bg-3βHSD1–5, pCold-Bg-3βHSD7, and pCold-Bg-HSD3B7 constructs. These constructs were separately transformed into *Escherichia coli* BL21 CondonPlus (DE3)-RIPL, and positive colonies were selected on LB agar containing ampicillin (100 μg/ml) and chloramphenicol (25 μg/ml) to get individual recombinant *E. coli* strains including BL-pCold-Bg-3βHSD1–5, BL-pCold-Bg-3βHSD7, and BL-pCold-Bg-HSD3B7. All positive clones were confirmed by PCR and sequencing (Sangon Biotech, Shanghai).

### Enzyme activity assay

Crude enzyme extracts from individual recombinant *E. coli* strains were separately prepared for *in vitro* enzyme activity assays. Positive colonies were inoculated (5%, v/v) into LB medium and induced at OD_600_ = 0.6–0.8 with 0.5 mM IPTG at 15°C for 24 h. Cells were harvested (4000×***g***, 20 min, 4°C), resuspended in PBS (20 mM, pH 7.4), lysed by sonication on ice, and centrifuged (12,000×***g***, 20 min, 4°C) to obtain crude enzyme extracts. Protein concentration was determined by the Bradford assay following the protein standard curve generated using bovine serum albumin as the standard (Supplementary Figure S1).

Steroid substrates were dissolved in 95% ethanol to prepare 15 mM stock solutions. NADH and NADPH were dissolved in 10 mM NaOH to a final concentration of 50 mM, whereas NAD^+^ and NADP^+^ were prepared in water at 50 mM. Reaction mixtures (150 μl) consisted of 75 μl crude enzyme extract and 75 μl buffer containing 0.6 mM substrate and 2 mM cofactor. Reactions were incubated at 30°C for 2 h. Reactions were stopped by the addition of ethanol, filtered (0.45 μm), and stored for analysis. Substrate conversion efficiency was calculated using the peak area (*A* experimental sample or *A* control sample) in high-performance liquid chromatography (HPLC) profiles as follows:

$$\text{Conversion}\;\;\;\text{efficiency}\;(\%)=\left[1-\frac{A\;\;\text{experimental}\;\text{sample}}{A\;\;\text{control}\;\text{sample}}\right]\times 100\%$$

For substrates and products with authentic standards available, oxidation reactions with NAD^+^ or NADP^+^ and reduction reactions with NADH or NADPH were performed separately, and conversion efficiencies were determined based on the corresponding standards. For reactions yielding putative products without authentic standards, NAD^+^ and NADP^+^ (for oxidation reactions) or NADH and NADPH (for reduction reactions) were supplied as mixed cofactors. Enzyme activity and conversion efficiencies were evaluated using these combined cofactor systems based on product detection, due to the lack of authentic standards for quantitative calibration.

### Recombinant Bg-3βHSD2 protein purification

Recombinant Bg-3βHSD2 protein expressed in *E. coli* was purified using Ni-NTA affinity chromatography. The purification conditions were as follows:

Cells were resuspended in enhanced lysis buffer (20 mM phosphate buffer, 500 mM NaCl, 10% glycerol, 1 mM DTT, pH 7.4) and disrupted by ultrasonication (3 mm probe, 33% amplitude, ∼180 W, 2 s/5 s off cycles for 20 min on ice, maintaining temperature below 16°C). The lysate was centrifuged at 12,000×***g*** for 30 min at 4°C, and the supernatant was loaded onto a pre-equilibrated Ni-NTA column. Proteins were eluted using a buffer containing 150 mM imidazole (based on previously optimized conditions). All elution fractions were supplemented with 1 mM DTT and 10% glycerol.

The purified enzyme was concentrated using a 50 kDa molecular weight cutoff ultrafiltration device. To improve protein stability and preserve catalytic activity, the purified recombinant Bg-3βHSD2 protein was supplemented with NADPH immediately after purification, as described previously [24].

### Effects of pH and temperature on recombinant Bg-3βHSD2 activity

The effects of pH and temperature on recombinant Bg-3βHSD2 activity were evaluated using crude enzyme extracts and the standard reaction system described above. Epipregnanolone (**5**) was used as a representative substrate for all optimization experiments. For temperature optimization, reactions were incubated at 20–50°C for 0.5 h at pH 7.4. For pH optimization, assays were performed at 30°C for 0.5 h using 0.1 M buffers, including PBS (pH 6.0–7.5), Tris–HCl (pH 7.1–9.0), and glycine-NaOH (pH 8.6–10.0). Only pH and temperature were varied, while other reaction conditions were kept constant.

Product formation (5β-pregnane-3,20-dione(**6**) was analyzed by HPLC. Quantification was performed using a calibration curve generated with dihydroprogesterone as an external standard. Briefly, a 5 mg/ml dihydroprogesterone stock solution was prepared and serially diluted to construct the standard curve (*y* = 12.535*x* + 0.1909, *R*^2^ = 0.9993; 9.765–1250 μg/ml) (Supplementary Figure S2).

### Liquid chromatography–tandem mass spectrometry analysis

Target metabolomic assay was performed to quantify major bufadienolides, including resibufogenin, cinobufagin, bufalin, telocinobufagin, and cinobufotalin, in different tissues of toad samples using liquid chromatography–tandem mass spectrometry (LC-MS/MS). Tissue samples were extracted with 95% ethanol, concentrated under nitrogen, reconstituted in methanol, and filtered through a 0.22 μm membrane. Analysis was performed on an Agilent 6410A triple quadrupole LC-MS/MS (Agilent Technologies, U.S.A.) with an ESI source in positive mode (dry gas was 350°C, 10 l/min; nebulizer was 40 psi). Chromatographic separation was achieved using a Waters XBridge-C18 column (100 × 2.1 mm, 3.5 μm) at 35°C, with 0.1% formic acid in water (mobile phase D) and acetonitrile (mobile phase C) at 0.3 ml/min under isocratic elution of 60% C/40% D for 4.5 min. The injection volume was 10 μl. Quantitation was conducted in MRM mode using the transitions as listed below (Table 2). Diphenhydramine (INN) was used as an internal standard reference.

**Table 2:** Optimized MRM parameters for quantitative analysis of bufadienolides

Molecules	Precursor ion (m/z)	Product ion (m/z)	Fragmentor voltage (V)	Collision energy (eV)	Ionization mode
Diphenhydramine	256.2	167.1	60	8	+
Resibufogenin	385.4	105.1	140	36	+
Cinobufagin	443.2	187.0	150	24	+
Bufalin	387.2	107.1	150	36	+
Telocinobufagin	403.4	349.2	150	20	+
Cinobufotalin	459.5	363.3	100	30	+

### High-performance liquid chromatography conditions

HPLC was employed to analyze enzymatic reaction products using a Thermo U3000 system (Thermo Fisher Scientific, U.S.A.) equipped with a charged aerosol detector (CAD) and a diode-array detector (DAD). Separation was performed on a Phenomenex Luna column (250 × 4.6 mm, 5 μm) at 30°C. CAD data were acquired at 10 Hz with a nebulizer temperature of 45°C, and DAD detection was conducted at 205, 240, and 296 nm. The injection volume was 20 μl. Four substrate-specific methods were established as follows:

For hormones, mobile phases consisted of acetonitrile (A) and water (B). The gradient was 65%–76% A (0–5 min), 76%–80% A (5–13 min), and 80%–95% A (13–15 min), at a flow rate of 0.8 ml/min.

For sterols and oxysterols, mobile phases were A/B. The gradient was 75%–85% A (0–10 min, flow 0.5–1.0 ml/min), 85%–100% A (10–15 min, flow up to 1.5 ml/min), followed by isocratic 100% A (15–50 min, 1.5 ml/min).

For bile acids, mobile phases were acetonitrile (A) and 0.1% formic acid in water (C). The gradient was 48%–60% A (0–20 min), 60%–90% A (20–25 min), and 90%–100% A (25–34 min), at a flow rate of 0.8 ml/min.

For bufadienolides, mobile phases were acetonitrile (A) and 20 mM ammonium acetate with 0.3% acetic acid (C). The gradient was 25% A (0–10 min), 40% A (10–25 min), and 95% A (25–40 min), with a flow rate of 1.2 ml/min.

## Results

### Transcriptomic data assembly and functional annotation

A total of 63.9 Gb of transcriptomic sequencing data was generated, including 30.7 and 33.2 Gb of high-quality reads from the control and Pb^2+^-treated groups, respectively (BioProject accession number: PRJNA642325). After quality filtering, 91.70% of bases reached Q30 (error rate <0.1%), indicating that the majority of reads were of sufficient quality for reliable downstream assembly. A total of 183,776 unigenes with an average length of 793.59 bp were obtained from the transcriptomic databases of the parotoid gland, skin, liver, and adrenal gland tissues of two *B. bufo gararizans* Cantor ecotypes (Supplementary Figure S3A). Length distribution showed the highest proportion of unigenes in the 0–500 bp range, while those >1000 bp accounted for 18% (32,165) and 57% (105,407), respectively (Supplementary Figure S3B).

**Figure 1:**
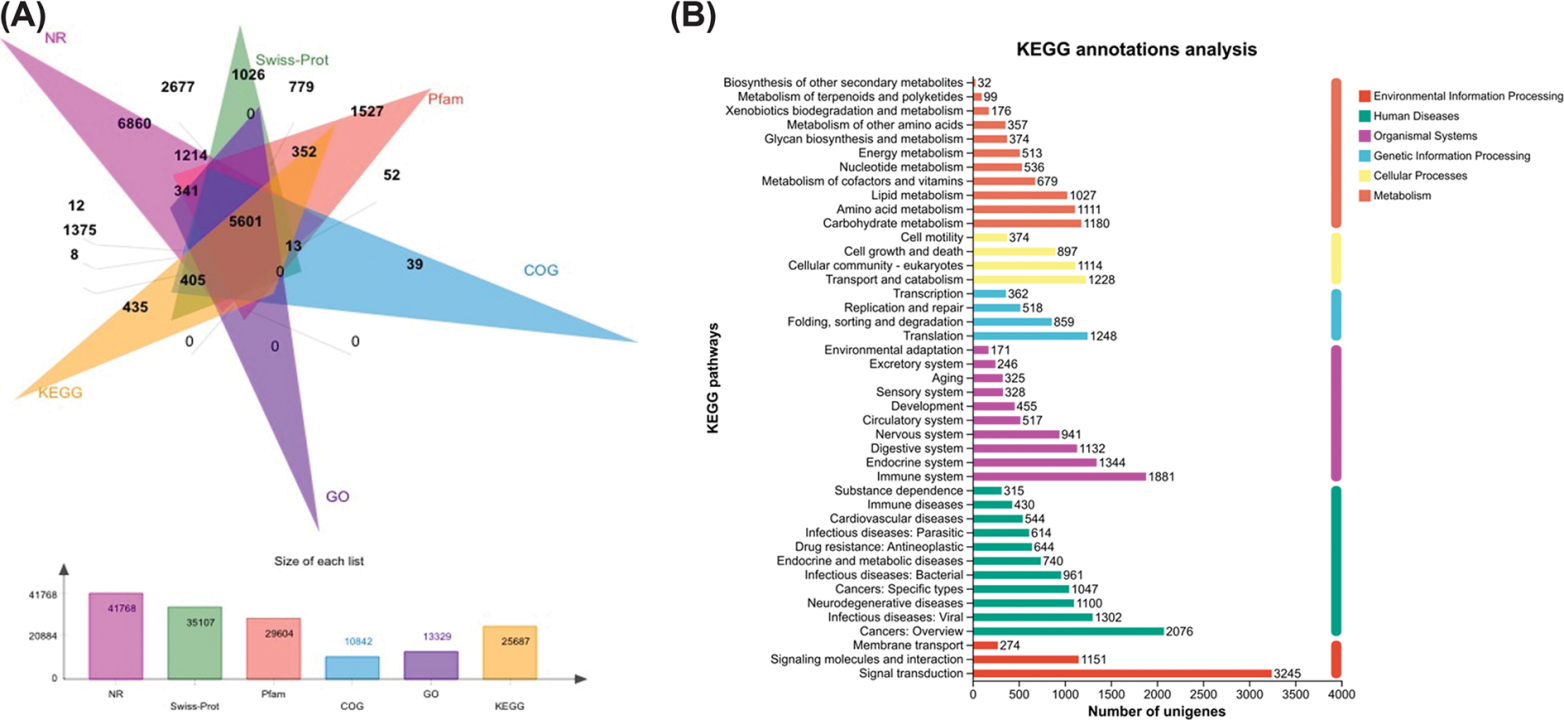
Functional annotation and classification of assembled unigenes from *B. bufo gargarizans* transcriptome (**A**) Overlap of unigene annotations among six public databases (NR, Swiss-Prot, Pfam, COG, GO, and KEGG). Numbers within the diagram indicate the number of unigenes shared among different databases. The bar chart below shows the total number of unigenes annotated in each database. (**B**) KEGG functional classification of assembled unigenes. The y-axis shows KEGG pathway categories, and the x-axis indicates the number of annotated unigenes assigned to each category.

Using the BLAST algorithm, all assembled unigenes were compared against six protein databases (NR, Swiss-Prot, Pfam, COG, GO, and KEGG) to assess sequence similarity (Figure 1A). The BLAST results demonstrated broad transcriptome coverage for *B. bufo gargarizans*, with 47,758 unigenes (25.99%) receiving functional annotation (Supplementary Figure S3C). The NR database contributed the largest number of annotations, with 41,768 unigenes (22.73%) (Figure 1A and Supplementary Figure S3C). A total of 5601 unigenes were co-annotated across all six databases, providing high-confidence functional assignments for a substantial subset of the transcriptome (Figure 1A). Among the NR-annotated unigenes, those exhibiting 80%–100% sequence identity accounted for the highest proportion (33.86%). Species distribution based on NR annotations showed that the majority of unigenes matched *Xenopus tropicalis* (18,944 unigenes), followed by Xenopus laevis (3173 unigenes) (Supplementary Figure S3D).

To characterize the metabolic pathways in *B. bufo gargarizans*, KEGG mapping was performed for 183,776 unigenes. A total of 25,687 functionally annotated unigenes were assigned to 320 KEGG pathway branches. According to the KEGG database classification, annotated unigenes were grouped into six major metabolic categories encompassing 43 subcategories, among which 4,685 unigenes were aligned to the ‘Metabolism’ category. Within all secondary metabolic pathways, the ‘Steroid hormone biosynthesis’ pathway (ko00140) contained the largest unigene cluster (224 members), followed by ‘Primary bile acid biosynthesis’ (ko00120; 151 members) and ‘N-glycan biosynthesis’ (ko00510; 83 members) (Figure 1B).

### Unigenes in steroid biosynthesis

Unigenes related to the ‘terpenoid backbone biosynthesis’ (ko00900) and ‘steroid biosynthesis‘ (ko00100) pathways were identified from the transcriptomic datasets. Except for 2-C-methyl-D-erythritol 2,4-cyclodiphosphate synthase, all enzymes of the MVA pathway and those related to cholesterol biosynthesis including acetoacetyl-CoA thiolase (AACT), squalene synthase, NAD(P)-dependent steroid dehydrogenase-like, and lanosterol synthase were annotated (Supplementary Table S4). These enzymes were encoded by varying numbers of unigenes, with AACT represented by the highest copy number, with 14 unigenes (Supplementary Table S4).

Following the formation of cholesterol, additional enzyme-mediated modifications such as hydroxylation, epoxidation, acetylation, glycosylation, and ketone formation are required to form bufadienolides. Potential candidate genes encoding the relevant tailoring enzymes include dehydrogenases, cytochrome P450 enzymes (CYP450s), and acyltransferases [19,20,25–27]. Transcriptome annotation revealed the presence of multiple members of these enzyme families in *B. bufo gargarizans*. Among the identified dehydrogenase-related genes, seven putative 3βHSD genes were selected for further characterization in the present study.

### Identification and bioinformatics analysis of Bg-3βHSDs

Given the importance of 3βHSD in steroid metabolism and bufadienolide biosynthesis, seven annotated *3βHSD* genes (*Bg-3βHSD1–7*) were selected from the transcriptome database for functional study. To extend the 3βHSD repertoire in *B. bufo gargarizans*, an additional gene, *Bg-HSD3B7* (GenBank accession no. XM 044303756.1), was also retrieved from GenBank for functional assays.

Bioinformatics analysis revealed that each gene contains the conserved Pfam-defined 3βHSD domain. Their open reading frames ranged from 861 to 1323 bp, encoding proteins of 286–440 amino acids (33.1–50.0 kDa) with predicted instability indices from 34.46 to 46.15 (Supplementary Table S5). Sequence identity among the eight Bg-3βHSDs varied from 12.7% to 51.5%, with Bg-3βHSD1 and Bg-3βHSD6 sharing the highest similarity (Supplementary Table S6).

**Figure 2:**
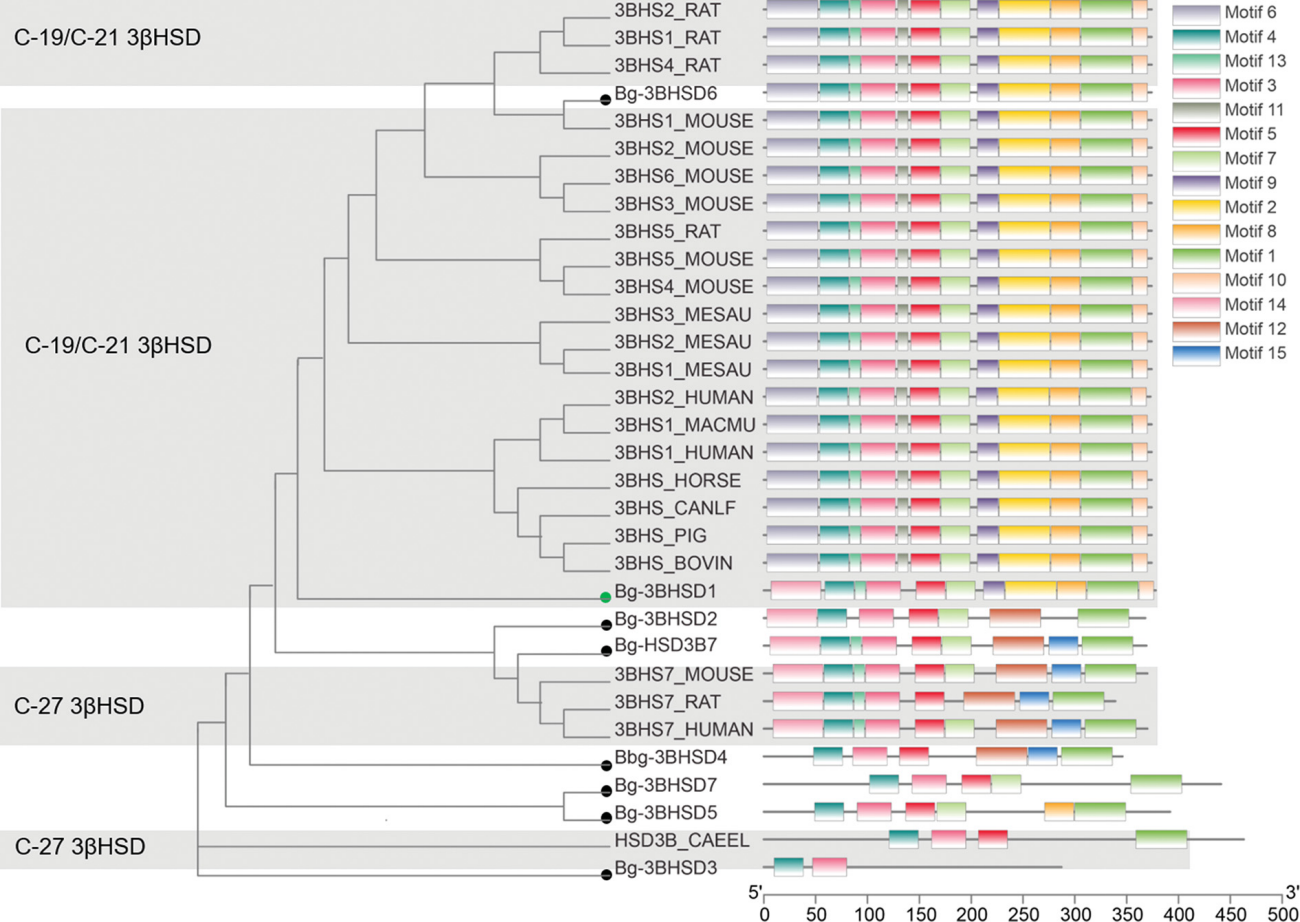
Phylogenetic relationships and conserved motif distributions of Bg-3βHSD proteins and representative 3βHSD homologs The phylogenetic tree was reconstructed using the ML method implemented in MEGA X with 1000 bootstrap replicates. The dataset includes Bg-3βHSD1, Bg-3βHSD2, Bg-3βHSD3, Bg-3βHSD-4, Bg-3βHSD5, Bg-3βHSD6, Bg-3βHSD7, Bg-HSD3B7, and representative 3βHSD homologs from other species. Experimentally characterized 3βHSD enzymes are highlighted in grey. Bg-3βHSD1, Bg-3βHSD2, Bg-3βHSD3, Bg-3βHSD4, Bg-3βHSD5, Bg-3βHSD6, Bg-3βHSD7, and Bg-HSD3B7 are indicated by a solid dot preceding their names in the phylogenetic tree. Conserved motifs identified by MEME are displayed to the right of each protein sequence, with different colored boxes representing distinct motifs. Bg-3βHSD1 represents the previously reported 3βHSD from *B. bufo gargarizans*, and Bg-3βHSD3B7 was identified from the transcriptome dataset generated in the present study.

Phylogenetic analysis along with the reported 3βHSD members (Supplementary Table S7) revealed that Bg-3βHSD2, 3, 4, 5, 7, and BgHSD3B7 grouped with C27-type steroid-metabolizing enzymes (e.g., 3BHS7_MOUSE, 3BHS7_RAT, 3BHS7_HUMAN, HSD3B_CAEEL), while Bg-3βHSD1 and Bg-3βHSD6 clustered with C19/C21-type catalytic homologs (e.g., 3BHS2_HUMAN, 3BHS2_MESAU, 3BHS1_MOUSE) (Figure 2). e dataset generated in the present study.

### Transcription profiles of *Bg-3βHSD* transcripts

**Figure 3:**
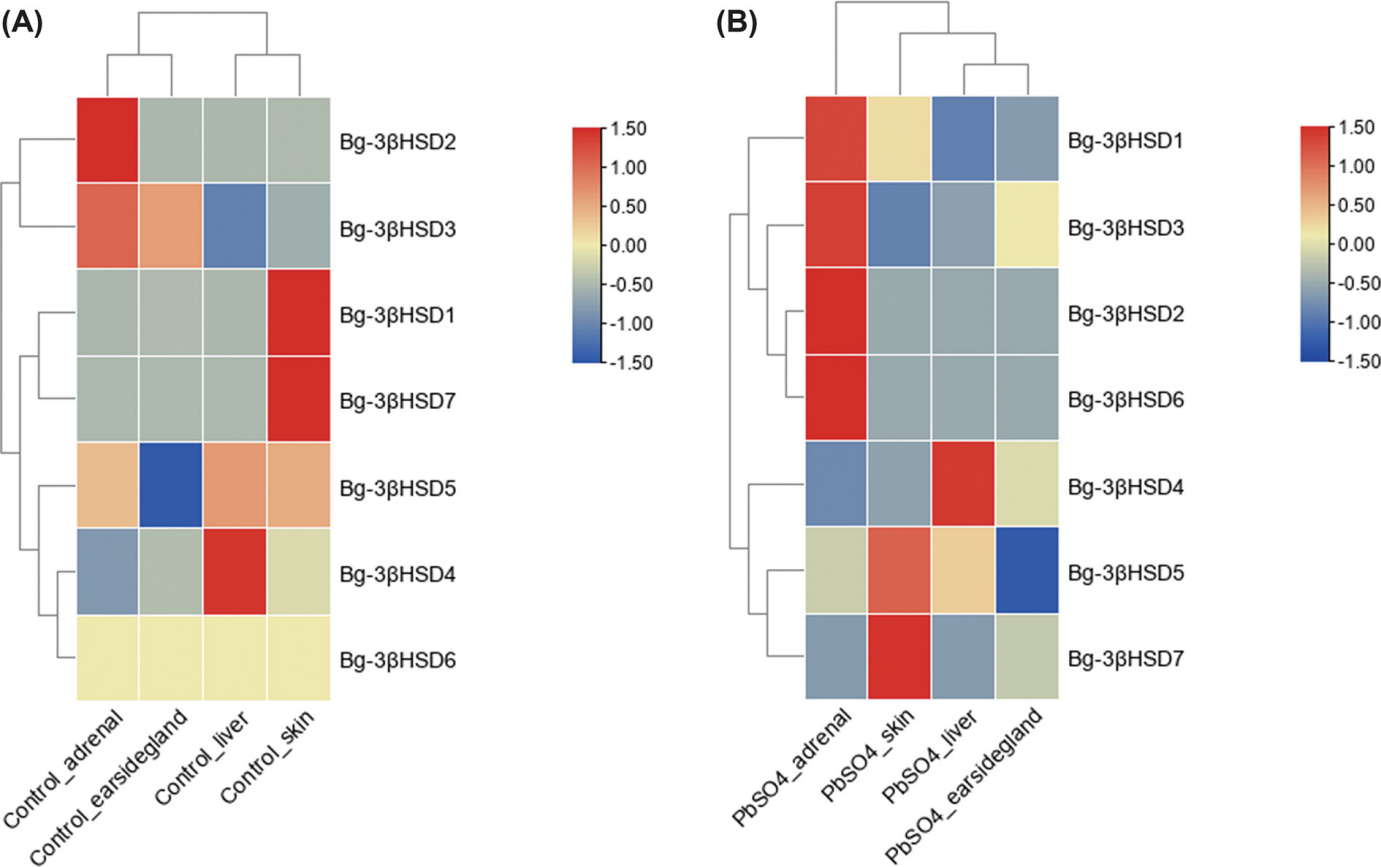
Heatmap of *Bg-3βHSD* transcript expression (**A**) Control samples. (**B**) PbSO_4_-treated samples.

The transcriptional profiles of *Bg-3βHSD1–7* transcripts across *B. bufo gargarizans* tissues were analyzed using the transcriptomic data under control and PbSO_4_-treated conditions. All genes displayed tissue-specific or tissue-enriched expression patterns (Figure 3 and Supplementary Figure S4). In the control group, *Bg-3βHSD1* showed a high expression level in skin and the parotoid gland, while *Bg-3βHSD2* was expressed specifically in the adrenal gland. *Bg-3βHSD3*, *Bg-3βHSD4*, and *Bg-3βHSD5* were broadly expressed across the liver, skin, parotoid, and adrenal gland. *Bg-3βHSD6* exhibited no appreciable expression in any of the four tissues, whereas *Bg-3βHSD7* was expressed in skin (Figure 3A).

After Pb^2+^ exposure, *Bg-3βHSD1* was strongly induced in the adrenal and parotoid glands. *Bg-3βHSD2* was predominantly expressed in the adrenal gland but showed minimal Pb^2+^ responsiveness. *Bg-3βHSD3*, *4*, and *5* were constitutively expressed across all tissues with little change under Pb^2+^ treatment, among which *Bg-3βHSD3* showed the highest transcript abundance. *Bg-3βHSD6* transcripts were induced in the adrenal gland upon PbSO_4_ exposure (Figure 3B and Supplementary Figure S4). *Bg-3βHSD7* was markedly up-regulated in the parotoid gland following Pb^2+^ treatment. These genes were identified as significantly differentially expressed genes (*P*adj <0.05, |log_2_FC| ≥1).

These patterns indicate functional divergence among these Bg-3βHSDs. Bg-3βHSD1, Bg-3βHSD2, Bg-3βHSD6, and Bg-3βHSD7 are likely involved in stress-responsive steroid or bufadienolide metabolism, whereas Bg-3βHSD3, Bg-3βHSD4, and Bg-3βHSD-5 contribute to basal steroid metabolism. Correlation analysis with their transcriptional profiles supported these distinctions. In controls, the expression of *Bg-3βHSD1* positively correlated with that of *Bg-3βHSD7*, while the expression of *Bg-3βHSD3* negatively correlated with that of *Bg-3βHSD4* (*r* = 1.000, *P*<0.05) (Supplementary Table S8). Under Pb^2+^ treatment, *Bg-3βHSD6* expression showed strong positive correlations with *Bg-3βHSD1–3* expression, and *Bg-3βHSD2* expression exhibited a strong positive correlation with the expression of *Bg-3βHSD3* (*r* = 1.000, *P*<0.05) (Supplementary Table S9).

### Quantitative assay of main bufadienolides and correlation analysis with *Bg-3βHSDs*

**Figure 4:**
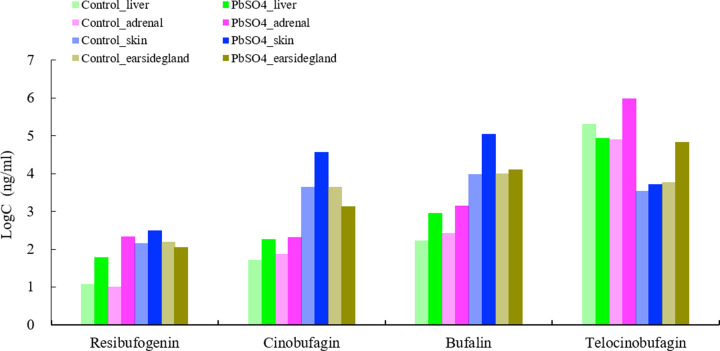
Content of bufadienolides in different tissues of *B. bufo gargarizans*

As shown in Figure 4, the concentrations of resibufogenin, cinobufagin, bufalin, telocinobufagin, and cinobufotalin exhibited distinct tissue-specific profiles. Telocinobufagin was the most abundant bufadienolide across all four tissues, followed by bufalin, cinobufagin, and resibufogenin. After Pb^2+^ exposure, telocinobufagin levels increased in adrenal, skin, and parotoid glands, but decreased in liver. Pb^2+^ treatment also elevated the levels of resibufogenin, cinobufagin, and bufalin in skin, liver, and adrenal glands, with the most pronounced increase occurring in skin. In contrast, resibufogenin and cinobufagin were reduced in parotoid glands upon Pb^2+^ exposure, and bufalin showed only a slight increase. Cinobufotalin was not detected in any tissues from either the control (Bg) or Pb^2+^-treated (Cg) groups. These results indicated that bufadienolides are synthesized or accumulated in all four examined tissues, and that their synthesis or accumulation is influenced by environmental stressors, such as Pb^2+^ exposure.

Correlation analysis revealed that these four bufadienolides were significantly correlated with each other in both control and Pb^2+^-treated samples, suggesting that their biosynthesis is tightly interconnected. Analysis of transcript and metabolite associations showed that *Bg-3βHSD7* expression was positively correlated with the levels of all four bufadienolides in the Pb^2+^-treated group (Supplementary Tables S8 and S9), suggesting a potential involvement of Bg-3βHSD7 in bufadienolide biosynthesis. No significant positive correlations were observed between the expression of the other *Bg-3βHSDs* and bufadienolide levels. However, these correlation patterns should be interpreted cautiously, as transcript abundance does not necessarily reflect enzyme activity due to potential post-transcriptional and post-translational regulation. In addition, metabolite transport and tissue compartmentalization in animals may further obscure direct relationships between gene expression and metabolite accumulation.

### Functional characterization of Bg-3βHSDs

**Figure 5:**
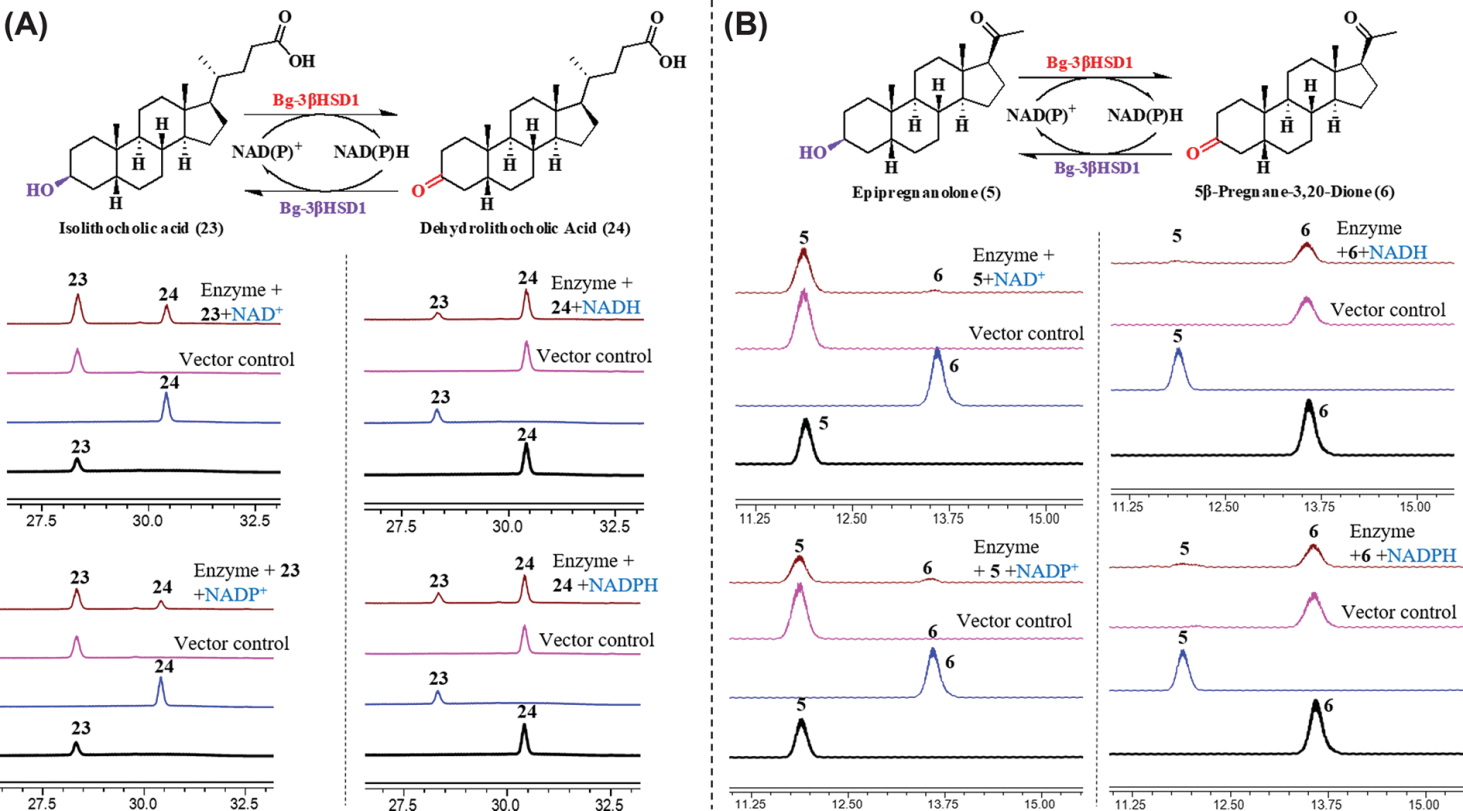
Representative HPLC chromatograms showing the redox activity of recombinant Bg-3βHSD1 enzyme toward steroid substrates Redox conversion of isolithocholic acid (**23**) and dehydrolithocholic acid (**24**) (**A**), and epipregnanolone (**5**) and 5β-pregnane-3,20-dione (**6**) (**B**), catalyzed by the recombinant Bg-3βHSD1 enzyme. Oxidation reactions were performed using NAD^+^ or NADP^+^ as a cofactor, whereas reduction reactions were performed using NADH or NADPH as a cofactor. In each chromatogram, traces are arranged from bottom to top as follows: authentic substrate standard, authentic product standard, reaction mixture containing crude extracts from *E. coli* cells harboring the empty pCold vector (vector control), and reaction mixture containing crude extracts from *E. coli* cells expressing recombinant Bg-3βHSD1 together with the indicated substrate and cofactor. Compound numbers correspond to those assigned in Table 1 and are indicated in the reaction schemes above the corresponding chromatograms.

Seven of the eight *Bg-3βHSD* genes were successfully cloned and expressed in *E. coli*. The *Bg-3βHSD6* transcript could not be obtained and was therefore excluded from subsequent functional characterization. SDS–PAGE analysis showed that all recombinant proteins were expressed in soluble form (Supplementary Figure S5A). Densitometric analysis indicated that recombinant Bg-3βHSD proteins accounted for approximately 9.90%–39.26% of the total soluble proteins (Supplementary Figure S5A). *In vitro* enzymatic assay demonstrated that recombinant Bg-3βHSD1, Bg-3βHSD2, and Bg-HSD3B7 enzymes displayed clear catalytic activities toward steroid substrates (Figure 5, Supplementary Figures S6–S13, and Table 3).

**Table 3:** Substrate specificity, cofactor utilization, and conversion efficiency of recombinant Bg-3βHSD enzymes toward steroid substrates

Recombinant Enzymes	Substrates	Products	Nucleotide Substrate	Conversion Efficiency	Typical HPLC Profile Ref.
Bg-3βHSD1	Isolithocholic acid (**23**)	Dehydrolithocholic acid (**24**)	NAD^+^	33.33%	Figure 5A
			NADP^+^	23.61%	
	Dehydrolithocholic acid (**24**)*	Isolithocholic acid (23)*	NADH	19.34%	
			NADPH	27.31%	
	5β-Pregnane-3,20-Dione (**6**)*	Epipregnanolone (**5**)*	NADH	11.94%	Figure 5B
			NADPH	17.38%	
	Epipregnanolone (**5**)	5β-Pregnane-3,20-Dione (**6**)	NAD^+^	4.1%	
			NADP^+^	9.95%	
	Dehydroepiandrosterone (**9**)	Androstenedione (**10**)	NAD^+^	1.75%	Supplementary Figure S6A
			NADP^+^	0.86%	
	Pregnenolone (**11**)	Progesterone (**12**)	NAD^+^	0.86%	Supplementary Figure S6B
			NADP	1.12%	
Bg-3βHSD2	Bufotaline (**32**)	3-oxo-Bufotaline (**48**)^#^	NAD^+^ & NADP^+^	90.22%	Supplementary Figure S7
	Resibufogenin (**36**)	3-oxo-Resibufogenin (**52**)^#^	NAD^+^ & NADP^+^	89.40%	Supplementary Figure S7
	Bufalin (**31**)	3-oxo-Bufalin (**47**)^#^	NAD^+^ & NADP^+^	80.97%	Supplementary Figure S7
	Arenobufagin (**33**)	3-oxo-Arenobufagin (**49**)^#^	NAD^+^ & NADP^+^	78.99%	Supplementary Figure S7
	Desacetylcinobufagin (**38**)	3-oxo-Desacetylcinobufagin (**54**)^#^	NAD^+^ & NADP^+^	72.93%	Supplementary Figure S7
	Cinobufagin (**37**)	3-oxo-Cinobufagin (**53**)^#^	NAD^+^ & NADP^+^	56.88%	Supplementary Figure S7
	Telocinobufagin (**35**)	3-oxo-Telocinobufagin (**51**)^#^	NAD^+^ & NADP^+^	9.05%	Supplementary Figure S7
	**Androstenediol (7)**	**Dehydroepiandrosterone (9)**	NAD^+^	76.95%	Supplementary Figure S8A
			NADP^+^	86.74%	
	Epipregnanolone (**5**)	5β-Pregnane-3,20-Dione (**6**)	NAD^+^	61.39%	Supplementary Figure S9A
			NADP^+^	71.34%	
	Allopregnanolone (**13**)	5α-Pregnane-3,20-dione (**41**)^#^	NAD^+^ & NADP^+^	45.67%	Supplementary Figure S10A
	Epiandrosterone (**1**)	5α-Androstanedione (**2**)	NAD^+^	42.65%	Supplementary Figure S11A
			NADP^+^	20.34%	
	**3β-Androstanediol (3)**	**Epiandrosterone (1)**	NAD^+^	26.93%	Supplementary Figure S11B
			NADP^+^	39.75%	
	**Testosterone (8)**	**Androstenedione (10)**	NAD^+^	36.88%	Supplementary Figure S8C
			NADP^+^	13.41%	
	5β-Pregnane-3,20-Dione (**6**)*	Epipregnanolone (**5**)*	NADH	31.49%	Supplementary Figure S9A
			NADPH	17.77%	
	Dehydroepiandro-sterone (**9**)	Androstenedione (**10**)	NAD^+^	6.87%	Supplementary Figure S8B
			NADP^+^	0.61%	
	Androstanolone (**4**)*	Androstanediol (**3**)*	NADH	2.06%	Supplementary Figure S11C
			NADPH	0.35%	
	Pregnenolone (**11)**	Progesterone (**12**)	NAD^+^	1.58%	Supplementary Figure S9B
			NADP^+^	0.24%	
	Isolithocholic acid (**23**)	Dehydrolithocholic acid (**24**)	NAD^+^	66.34%	Supplementary Figure S12A
			NADP^+^	84.19%	
	Dehydrolithocholic acid (**24**)*	Isolithocholic acid (**23**)*	NADH	28.25%	Supplementary Figure S12B
			NADPH	31.63%	
	7,12-Dihydroxy-3-oxocholanic acid (**29**)	3β-Cholic acid (**42**)^#^	NADH &NADPH	12.26%	Supplementary Figure S10B
Bg-HSD3B7	7α-Hydroxycholesterol (**16**)	7α-Hydroxycholest-4-en-3-one (**17**)	NAD^+^	20.12%	Supplementary Figure S13A
			NADP^+^	9.80%	
	7α,27-Dihydroxycholesterol (**22**)	7α,26-Dihydroxycholest-4-en-3-one (**45**)^#^	NAD^+^ & NADP^+^	17.74%	Supplementary Figure S13B

**Note:** Standard reaction mixtures (150 μl) consisted of 75 μl crude enzyme extract and 75 μl reaction buffer containing 0.6 mM steroid substrate and 2 mM cofactor. Reactions were carried out at pH 7.4 and 30°C for 2 h. Compounds highlighted in bold are involved in oxidation at the C17 position, and those marked with an asterisk (*) undergo reduction at the C3 position during conversion to the corresponding products. Products marked with a hash symbol (#) are putative metabolites inferred from the established catalytic mechanism of 3βHSD enzymes. Conversion efficiencies were determined separately for individual cofactors (NAD^+^, NADP^+^, NADH, or NADPH) when authentic substrate and product standards were available. For reactions yielding putative products without authentic standards, mixed cofactor systems were used, and cofactor-specific conversion efficiencies were not determined.

The recombinant Bg-3βHSD1 exhibited bidirectional oxidation–reduction activity toward bile acids, including isolithocholic acid (**23**) and dehydrolithocholic acid (**24**), with a conversion efficiency of approximately 30% in both directions (Figure 5A). It also catalyzed the redox conversion between the hormone substrates epipregnanolone (**5**) and 5β-pregnane-3,20-dione (**6**) (Figure 5B), and exhibited oxidative activity converting dehydroepiandrosterone (**9**) to androstenedione (**10**) and pregnenolone (**11**) to progesterone (**12**) (Supplementary Figure S6). The observed oxidation of **9** and **11** is consistent with the previously reported function of this enzyme [20].

The recombinant Bg-3βHSD2 enzyme exhibited high catalytic efficiency and broad substrate specificity. It could accept hormones, bile acids, and bufadienolactones as substrates (Supplementary Figures S7–S12), achieving a conversion rate of up to 90.22% for bufotaline (**32**) to the putative 3-oxo-bufotalin (**48**) (Table 3). It efficiently oxidized resibufogenin (**36**), bufalin (**31**), arenobufagin (**33**), and desacetylcinobufagin (**38**) to their respective 3-oxo derivatives (conversion >70%), and showed strong oxidative activity toward androstenediol (**7**, 86.74%), isolithocholic acid (**23**, 84.19%), and epipregnanolone (**5**, 71.34%), followed by allopregnanolone (**13**), epiandrosterone (**1**), androstanediol (**3**), and testosterone (**8**). This enzyme also exhibited considerable reductive activities toward dehydrolithocholic acid (**24**), 5β-pregnane-3,20-dione (**6**), and androstanolone (**4**) to their corresponding C3-reduced products. It also catalyzed the C3-oxidation and △^5^→△^4^ isomerization of dehydroepiandrosterone (**9**) and pregnenolone (**11**).

Notably, Bg-3βHSD2 exhibited C17 oxidation activity toward multiple hormone substrates, converting 3β-androstanediol (**3**) to epiandrosterone (**1**) (Supplementary Figure S11B), androstenediol (**7**) to dehydroepiandrosterone (**9**) and subsequently to androstenedione (**10**) (Supplementary Figure S8A,B), and testosterone (**8**) to androstenedione (**10**) (Supplementary Figure S8C). For the sequential conversion of androstenediol (**7**), dehydroepiandrosterone (**9**) accumulated as the major product, whereas only a small amount of androstenedione (**10**) was detected. Extension of the reaction time and supplementation with additional NAD^+^ or crude enzyme extracts resulted in only marginal changes in the formation of compound 10 (Supplementary Figure S14), suggesting limited further conversion of compound **9** under the assay conditions tested.

Interestingly, despite the presence of hydroxyl groups at both the C3 and C17 positions in androstenediol (**7**) and 3β-androstanediol (**3**), Bg-3βHSD2 preferentially oxidized the C17 hydroxyl group, with conversion efficiencies of 86.74% and 39.75% using NADP^+^ as cofactor, respectively (Table 3). These results reveal a pronounced preference of Bg-3βHSD2 for C17 hydroxyl oxidation over the canonical C3 reaction toward these hormone substrates.

Recombinant Bg-HSD3B7 exhibited catalytic activity toward C3 hydroxysteroid oxidation and Δ^5^→Δ^4^ double-bond isomerization of 7α-hydroxycholesterol (**16**) and 7α,27-dihydroxycholesterol (**22**) (Supplementary Figure S13 and Table 3), suggesting a potential role for Bg-HSD3B7 in the classical bile acid pathway associated with bufadienolide biosynthesis in toads. Nevertheless, further *in vivo* studies are required to establish its physiological role in this pathway.

To validate the catalytic activity of recombinant Bg-3βHSD enzymes, Bg-3βHSD2 was selected for purification (Supplementary Figure S5B) due to its broad substrate specificity and distinct catalytic profile among the active isoforms. As shown in Supplementary Figure S12C, the purified Bg-3βHSD2 retained catalytic activity toward dehydrolithocholic acid (**24**), converting it to isolithocholic acid (**23**) with conversion efficiencies of 18.93% and 22.80% using NADH and NADPH as cofactors, respectively. These values were slightly lower than those obtained with crude extracts expressing Bg-3βHSD2 (28.25% and 31.63%, respectively; Table 3). Furthermore, supplementation of the purified enzyme with crude extracts prepared from *E. coli* cells harboring the empty pCold vector did not result in a noticeable increase in catalytic activity (Supplementary Figure S12C). These results demonstrate that Bg-3βHSD2 is catalytically active in its purified form and that the observed activity in crude extracts is attributable to the recombinant enzymes.

Collectively, three catalytically active Bg-3βHSD enzymes, Bg-3βHSD1, Bg-3βHSD2, and Bg-HSD3B7, were identified. All three enzymes were able to utilize both NAD^+^/NADH and NADP^+^/NADPH as cofactors (Table 3).

### Activity profiles of Bg-3βHSD2 versus pH and temperature

**Figure 6:**
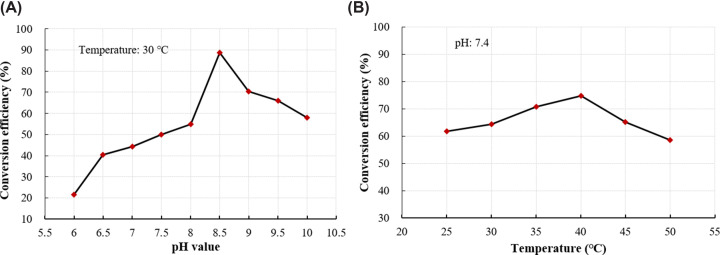
Effect of pH and temperature on the catalytic activity of recombinant Bg-3βHSD2 enzyme (**A**) pH-dependent activity profile of recombinant Bg-3βHSD2. (**B**) Temperature-dependent activity profile of recombinant Bg-3βHSD2.

The catalytic activity of recombinant Bg-3βHSD2 was evaluated under different pH and temperature conditions using epipregnanolone (**5**) as the substrate. The enzyme exhibited broad pH tolerance from 6.0 to 10.0, with maximal conversion (88.72%) observed at pH 8.5 (Figure 6A). Temperature-dependent assays showed that Bg-3βHSD2 retained catalytic activity between 25°C and 50°C, with activity increasing up to 40°C and then decreasing at higher temperatures. The highest conversion efficiency (74.81%) was observed at 40°C (Figure 6B). These results indicate that Bg-3βHSD2 is active under mildly alkaline conditions and retains activity over a relatively broad temperature range.

## Discussion

In the present study, we characterized multiple 3βHSD homologs from the Asiatic toad and examined their enzymatic activities against a panel of steroid substrates. Among the identified isoforms, Bg-3βHSD2 exhibited broad substrate specificity and distinct catalytic properties in comparison with other family members. These results expand our understanding of the functional diversity exhibited by 3βHSD enzymes in non-mammalian vertebrates.

3βHSDs catalyze the oxidation of Δ^5^-3β-hydroxysteroids to Δ^4^-3-ketosteroids and play an obligatory role in the biosynthesis of all classes of steroid hormones including progesterone, androgens, glucocorticoids, and mineralocorticoids, as well as their downstream metabolic pathways [1–7]. This catalytic activity is conserved across vertebrates and underpins essential steroidogenic processes. The expression and regulation of 3βHSD have been reviewed in mammals, where tissue-specific isoforms and multiple regulatory mechanisms have been described, indicating a complex genetic and biochemical control of steroid metabolism in different physiological contexts [28].

Previous research has shown that 3βHSD expression is not limited to classical steroidogenic organs but is also detectable in amphibian tissues, including the central nervous system, suggesting functional roles beyond canonical hormone production. For instance, studies in frogs have documented 3βHSD immunoreactivity in neural tissue, supporting the presence of steroidogenic activity in non-traditional locations in amphibians [29]. Our results further substantiate the notion that 3βHSD homologs in non-mammalian vertebrates can exhibit divergent functional properties, potentially contributing to species-specific steroid processing.

Bufadienolides represent a structurally diverse class of steroid-like compounds that are abundant in bufonid toads and contribute to their chemical defense strategies. These compounds contain the characteristic steroidal skeleton with modifications that distinguish them from classical vertebrate steroids, such as a lactone ring at the C-17 position [17]. The biosynthetic pathways leading to bufadienolide formation in toads remain incompletely defined, though earlier biochemical experiments have suggested that cholesterol and related steroids can serve as precursors to bufadienolides via yet-to-be-elucidated enzymatic steps [30].

Our data demonstrate that Bg-3βHSD2 possesses a broad catalytic profile, enabling the transformation of diverse steroid substrates *in vitro*, including intermediates of classical steroidogenic pathways and bufadienolide-related compounds. In addition to substrate promiscuity, Bg-3βHSD2 exhibits regioselective C17 oxidation activity, distinguishing it from canonical vertebrate 3βHSDs, which primarily catalyze C3 oxidation and Δ^5^→Δ^4^ isomerization. These distinctive catalytic characteristics are accompanied by substantial divergence at the sequence, structural, and expression levels, supporting the classification of Bg-3βHSD2 as a non-canonical 3βHSD with unique catalytic properties. The broad substrate range and atypical catalytic activity of Bg-3βHSD2 suggest that this enzyme may contribute to steroid metabolism in ways that differ from those of previously characterized vertebrate 3βHSDs [1,5]. However, further studies are required to determine the physiological significance and evolutionary origin of these properties.

**Figure 7:**
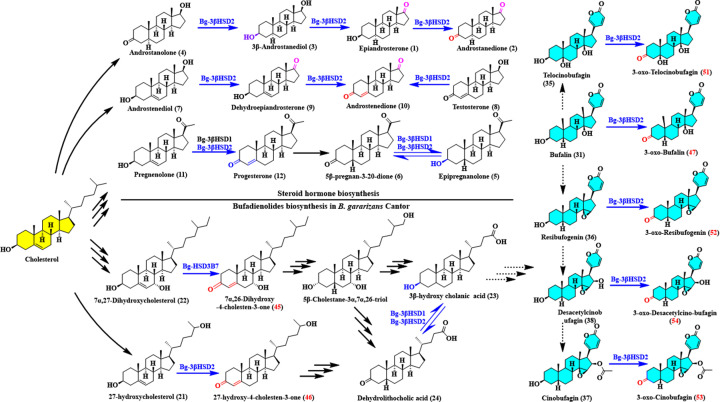
Bg-3βHSDs catalytic reactions in steroid metabolism Catalytic steps identified in the present study are highlighted in blue. Numbers in red represent predicted products inferred from the 3βHSD catalytic mechanism.

Although the precise biochemical routes of bufadienolide biosynthesis remain to be fully elucidated, our results suggest a potential involvement of 3βHSD enzymes in multiple steps of amphibian steroid metabolism (Figure 7). Given the structural diversity of bufadienolides and their metabolic precursors, the broad substrate range observed for Bg-3βHSD2 suggests functional versatility among steroid-transforming enzymes.

In this context, the identification of multiple putative downstream tailoring enzymes, including cytochrome P450s and acyltransferases (Supplementary Table S4), highlights the potential complexity of bufadienolide metabolism and provides a basis for future functional characterization. Together with the enzymatic properties of Bg-3βHSD2 described here, these findings suggest that bufadienolide diversity in *B. bufo gargarizans* may arise from the coordinated action of several steroid-modifying enzymes with partially overlapping substrate specificities. Further studies integrating targeted biochemical assays and *in vivo* functional analyses will be required to delineate the specific roles of individual enzymes within this metabolic network.

In conclusion, our analysis reveals that 3βHSD homologs from *B. bufo gargarizans* exhibit functional diversity, with Bg-3βHSD2 in particular displaying broad catalytic specificity. These findings contribute to a deeper understanding of steroid metabolism in amphibians and emphasize the need for future studies to delineate the metabolic networks linking classical steroidogenic enzymes to organism-specific metabolites like bufadienolides. The results presented here lay a foundation for such investigations and suggest that enzymatic flexibility within gene families may be a common feature in the evolution of specialized metabolic pathways.

## Supplementary Material

Supplementary Figures S1-S14 and Table S1-S9

## Data Availability

All data supporting the results of the present study are available within the article and its Supplementary Information files. Transcriptomic sequencing data have been deposited in the NCBI Sequence Read Archive under BioProject accession number PRJNA642325 [31]. The nucleotide sequences of Bg3βHSD1, Bg3βHSD2, Bg3βHSD3, Bg3βHSD4, Bg3βHSD5, Bg3βHSD6, Bg3βHSD7, and Bg-HSD3B7 have been deposited in GenBank under accession numbers MG517527 [32], MT328812 [33], MT328813 [34], MT328814 [35], MT328815 [36], MT328816 [37], MT328817 [38], and XM_044303756 [39], respectively.

## Abbreviations

3βHSD: 3β-hydroxysteroid dehydrogenase
AACT: acetoacetyl-CoA thiolase
CAD: charged aerosol detector
DAD: diode-array detector
HPLC: high-performance liquid chromatography
ML: maximum likelihood
LC-MS/MS: liquid chromatography–tandem mass spectrometry
NADH: nicotinamide adenine dinucleotide (reduced form)
NADPH: nicotinamide adenine dinucleotide phosphate (reduced form)
NADP^+^: nicotinamide adenine dinucleotide phosphate (oxidized form)
NAD^+^: nicotinamide adenine dinucleotide (oxidized form)
P450: cytochrome P450 monooxygenase
SDR: short-chain dehydrogenase/reductase
