# Supplementary material for: Functional characterization of 3β-hydroxysteroid dehydrogenases in Asiatic toad reveals an efficient broad substrate specificity of Bg-3βHSD2
Source: Biosci Rep. 2026 Aug 7;46(8):BSR20260032. doi: 10.1042/BSR20260032 (PMC13451435; doi:10.1042/BSR20260032)
Supplement: Supplementary file 1 [file BSR-2026-0032_supp.pdf]

Supplementary Materials

Functional characterization of 3β-hydroxysteroid dehydrogenases in Asiatic toad reveals broad substrate specificity of Bg-3βHSD2

Qian Meng[1], Yaoting Hu[1], Wenjie Chen[1], Chuanguang Xiao, Li Yang, Rufeng Wang, Beibei Zhang, Shujuan Zhao*, Zhengtao Wang

State Key Laboratory of Discovery and Utilization of Functional Components in Traditional Chinese Medicine, The SATCM Key Laboratory for New Resources & Quality Evaluation of Chinese Medicine, The MOE Key Laboratory for Standardization of Chinese Medicines and Shanghai Key Laboratory of Compound Chinese Medicines, Institute of Chinese Materia Medica, Shanghai University of Traditional Chinese Medicine, Shanghai 201203, China

[1]These two authors contributed equally to this work

*Corresponding author. E-mail address: zhaoshujuan@126.com, zhaoshujuan@shutcm.edu.cn. ORCID: 0000-0002-2269-925X

Table S1 Information of steroid compounds used in the study

No.	Classes	Name	CAS	Molecular formula	Manufacturer
**1**	Hormones	Epiandrosterone	481-29-8	$C_{19}H_{30}O_2$	Shanghai Macklin Biochemical Co., Ltd
**2**		Androstanedione	846-46-8	$C_{19}H_{28}O_2$	Shanghai Macklin Biochemical Co., Ltd
**3**		3β-Androstanediol	571-20-0	$C_{19}H_{32}O_2$	Shanghai Acmec Biochemical Co., Ltd
**4**		Androstanolone	521-18-6	$C_{19}H_{30}O_2$	Shanghai Acmec Biochemical Co., Ltd
**5**		Epipregnanolone	128-21-2	$C_{21}H_{34}O_2$	Shanghai Hongye Biotechnology Co., Ltd
**6**		5β-Pregnane-3,20-dione	128-23-4	$C_{21}H_{32}O_2$	Shanghai Macklin Biochemical Co., Ltd
**7**		Androstenediol	521-17-5	$C_{19}H_{30}O_2$	Shanghai Macklin Biochemical Co., Ltd
**8**		Testosterone	58-22-0	$C_{19}H_{28}O_2$	Shanghai Yuanye Bio-Technology Co., Ltd
**9**		Dehydroepiandrosterone	53-43-0	$C_{19}H_{28}O_2$	Shanghai Yuanye Bio-Technology Co., Ltd
**10**		Androstenedione	63-05-8	$C_{19}H_{26}O_2$	Shanghai Yuanye Bio-Technology Co., Ltd
**11**		Pregnenolone	145-13-1	$C_{21}H_{32}O_2$	Shanghai Aladdin Bio-Chem Technology Co., Ltd
**12**		Progesterone	57-83-0	$C_{21}H_{30}O_2$	Shanghai Yuanye Bio-Technology Co., Ltd
**13**		Allopregnanolone	516-55-2	$C_{21}H_{34}O_2$	Shanghai Chemegen Bio-Technology Co., Ltd.
**14**	Oxysterol	Cholesterol	57-88-5	$C_{27}H_{46}O$	Shanghai Macklin Biochemical Co., Ltd
**15**		4-Cholesten-3-One	601-57-0	$C_{27}H_{44}O$	Shanghai Yuanye Bio-Technology Co., Ltd
**16**		7α-Hydroxycholesterol	566-26-7	$C_{27}H_{46}O_2$	Guangzhou Haoromi Scientific Instrument Co., Ltd.
**17**		7α-Hydroxy-4-Cholesten-3-One	3862-25-7	$C_{27}H_{44}O_2$	Nanjing Beiyu Biotechnology Co., Ltd
**18**		β-Sitosterol	83-46-5	$C_{29}H_{50}O$	Sichuan Victory Biological Technology Co., Ltd.
**19**		Stigmasterin	83-48-7	$C_{29}H_{48}O$	Sichuan Victory Biological Technology Co., Ltd.
**20**		7β-Hydroxycholesterol	566-27-8	$C_{27}H_{46}O_2$	Sichuan Victory Biological Technology Co., Ltd.
**21**		27-Hydroxycholesterol	20380-11-4	$C_{27}H_{46}O_2$	Sichuan Victory Biological Technology Co., Ltd.
**22**		7α,27-Dihydroxycholesterol	4725-24-0	$C_{27}H_{46}O_3$	Shanghai Acmec Biochemical Co., Ltd
**23**	Bile acids	Isolithocholic acid	1534-35-6	$C_{24}H_{40}O_3$	Nanjing Beiyu Biotechnology Co., Ltd
**24**		Dehydrolithocholic Acid	1553-56-6	$C_{24}H_{38}O_3$	MedChemexpress
**25**		Chenodeoxycholic Acid	474-25-9	$C_{24}H_{40}O_4$	Shanghai Yien Chemical Technology Co., Ltd
**26**		Ursodeoxycholic Acid	128-13-2	$C_{24}H_{40}O_4$	Shanghai Yien Chemical Technology Co., Ltd
**27**		Lithocolic Acid	434-13-9	$C_{24}H_{40}O_3$	Shanghai Acmec Biochemical Co., Ltd
**28**		Cholic Acid	81-25-4	$C_{24}H_{40}O_5$	Shanghai Acmec Biochemical Co., Ltd

No.	Classes	Name	CAS	Molecular formula	Manufacturer
**29**		7,12-Dihydroxy-3-oxocholanic acid	2304-89-4	$C_{24}H_{40}O_4$	Shanghai Acmec Biochemical Co., Ltd
**30**		Desoxycholic Acid	83-44-3	$C_{24}H_{38}O_5$	Shanghai Acmec Biochemical Co., Ltd
**31**	Bufadienolides	Bufalin	465-21-4	$C_{24}H_{34}O_4$	Chengdu Pusi Biotechnology Co., Ltd.
**32**		Bufotaline	471-95-4	$C_{26}H_{36}O_6$	Chengdu Biopurify Phytochemicals Ltd.
**33**		Arenobufagin	464-74-4	$C_{24}H_{32}O_6$	Chengdu Pusi Biotechnology Co., Ltd.
**34**		Gamabufotalin	465-11-2	$C_{24}H_{34}O_5$	Chengdu Biopurify Phytochemicals Ltd.
**35**		Telocinobufagin	472-26-4	$C_{24}H_{34}O_5$	Chengdu Biopurify Phytochemicals Ltd.
**36**		Resibufogenin	465-39-4	$C_{24}H_{32}O_4$	Chengdu Pusi Biotechnology Co., Ltd.
**37**		Cinobufagin	470-37-1	$C_{26}H_{34}O_6$	Chengdu Pusi Biotechnology Co., Ltd.
**38**		Desacetylcinobufagin	4026-95-3	$C_{24}H_{32}O_5$	Chengdu Biopurify Phytochemicals Ltd.
**39**		Desacetylcinobufotalin	4099-30-3	$C_{24}H_{32}O_6$	Chengdu Biopurify Phytochemicals Ltd.
**40**		Cinobufotalin	1108-68-5	$C_{26}H_{34}O_7$	Shanghai Yuanye Bio-Technology Co., Ltd

Table S2 Information of coenzymes used in this study

Abbreviation	Name	CAS	Molecular formula	Manufacturer
NAD^+	β-Nicotinamide adenine dinucleotide trihydrate	53-84-9	$C_{21}H_{27}N_7O_{14}P_2$	Shanghai Sangon Biotech Co., Ltd.
NADH	β-Nicotinamide adenine dinucleotide disodium salt	606-68-8	$C_{21}H_{27}N_7Na_2O_{14}P_2$	Shanghai Sangon Biotech Co., Ltd.
$NADP^+$	β-Nicotinamide adenine dinucleotide phosphate disodium salt	24292-60-2	$C_{21}H_{26}N_7Na_2O_{17}P_3$	Beijing Coolaber Science & Technology Co., Ltd
NADPH	β-Nicotinamide adenine dinucleotide 2′- phosphate reduced tetrasodium salt	2646-71-1	$C_{21}H_{26}N_7Na_4O_{17}P_3$	Beijing Coolaber Science & Technology Co., Ltd

Table S3 Primers used in this study

Primer	Sequences (5'–3')	Enzyme site
Bg3βHSD1-F	CGGGGTACCATGAGCGGCGCGCTG	*Kpn*I
Bg3βHSD1-R	CCGCTCGAGCTCGAGTTTCTTTTTGTTTGCCTCCTTGCGTTG	*Xho*I
Bg3βHSD2-F	CGGGGTACCGCCACCATGGCGAAAGATCTGGTG	*Kpn*I
Bg3βHSD2-R	CCGCTCGAGTTTTTGTTTTAATTTTTCTTGACCTAGAATTTCC	*Xho*I
Bg3βHSD3-F	CGGGGTACCATGTATCTCCGACCTATGG	*Kpn*I
Bg3βHSD3-R	CCCAAGCTTGAAGTTCACAGGGGTGACT	*Hind*III
Bg3βHSD4-F	CGGGGTACCATGGCGACACGAATACG	*Kpn*I
Bg3βHSD4-R	CCGCTCGAGACTTCCCCTCCTCAGG	*Xho*I
Bg3βHSD5-F	CCGGAATTCATGGCTGCTCCACC	*EcoR*I
Bg3βHSD5-R	ACGCGTCGACTAGGACTAACCCCACCACCGTA	*Sal*I
Bg3βHSD6-F	CCGGGATCCATGGCTGGATGGAGCTGCCT	*BamH*I
Bg3βHSD6-R	ACGCGTCGACCTGGCACTTTGTGTCCAGTATCTC	*Sal*I
Bg3βHSD7-F	CCGGAATTCATGAGGGAAACTATGTCCGTATGTGAAC	*EcoR*I
Bg3βHSD7-R	ACGCGTCGACATGGTTCCATAGAAGCAAAGGTAGATACAAAAT	*Sal*I
Bg-HSD3B7-F	CCGGAATTCATGGCATCTGAGGAGGGGCAGGTGTA	*EcoR*I
Bg-HSD3B7-R	ACGCGTCGACTTGGCTCTTCGCCCCCTCCTCGAA	*Sal*I

Table S4 Unigenes encoding enzymes involved in Steroid biosynthesis in *B. bufo gargarizans*

Gene	KEGG Pathway	KEGG Entry	Enzyme	Enzyme Class	Reaction	Definition	Original Unigene ID (Local Identifier)	NCBI Gene ID (current *Bufo gargarizans* annotation)
AACT	ko00900	K00626	2.3.1.9	Transferases	R00238	Acetyl-CoA C-acetyltransferase	105485_c5_g1; 114895_c2_g1; 90769_c0_g4; 17543_c0_g1; 91607_c0_g31; 152349_c0_g1; 124913_c0_g1; 153813_c0_g1; 126786_c0_g1; 118366_c0_g1; 146908_c0_g1; 7722_c0_g1; 155064_c0_g1; 130342_c0_g1	122931107; 122934225
HMGS	ko00900	K01641	2.3.3.10	Transferases	R01978	Hydroxymethylglutaryl-CoA synthase	101113_c1_g1; 142126_c0_g1; 143646_c0_g1; 142317_c0_g1	122924776
HMGCR	ko00900	K00021	1.1.1.34	Oxidoreductases	R02082	Hydroxymethylglutaryl-CoA reductase	116236_c0_g3; 129079_c0_g1; 16483_c0_g1; 142973_c0_g1	122924719; 122924720; 122935929; 122935954; 122935962
MVK	ko00900	K00869	2.7.1.36	Transferases	R02245	Mevalonate kinase	98014_c10_g2; 98014_c10_g1	122933743
PMK	ko00900	K00938	2.7.4.2	Transferases	R03245	Phosphomevalonate kinase	146339_c0_g1	122932977
MVD	ko00900	K01597	4.1.1.33	Lyases	R01121	Diphosphomevalonate decarboxylase	93536_c3_g2; 145690_c0_g1	122922518
GGPS1	ko00900	K00804	2.5.1.1	Transferases	R01658	Geranylgeranyl diphosphate synthase, type III	103645_c3_g3	122934720
FDPS	ko00900	K00787	2.5.1.10	Transferases	R02003	Farnesyl diphosphate synthase	95103_c0_g1; 73848_c0_g1	122938034; 122934458
SQS	ko00100	K00801	2.5.1.21	Transferases	R00702	Farnesyl-diphosphate farnesyltransferase	107764_c6_g1; 115559_c2_g2; 92897_c6_g1; 102383_c1_g1; 115559_c2_g1; 92996_c10_g1;	122944545; 122944547; 122944548; 122944549;

							154825_c0_g1	122944550; 122944551; 122944552
SQLE	ko00100	K00511	1.14.14.17	Oxidoreductases	R02874	Squalene monooxygenase	103348_c0_g1; 137723_c0_g1	122937981
LSS	ko00100	K01852	5.4.99.7	Isomerases	R03199	Lanosterol synthase	113781_c3_g7	122944874; 122945066
CYP51	ko00100	K05917	1.14.14.154	Oxidoreductases	R05640	Sterol 14alpha-demethylase	91917_c0_g1; 81502_c0_g2	122938184
TM7SF2	ko00100	K00222	1.3.1.70	Oxidoreductases	R05639	Delta14-sterol reductase	114271_c6_g1; 122866_c0_g1	122920337
MESO1	ko00100	K07750	1.14.18.9	Oxidoreductases	R07509	Methylsterol monooxygenase	92533_c3_g1; 114603_c7_g2	122940276; 122940341
NSDHL	ko00100	K07748	1.1.1.170	Oxidoreductases	R07494	Sterol-4alpha-carboxylate 3-dehydrogenase (decarboxylating)	105027_c0_g1; 120681_c0_g1; 131305_c0_g1	122946536
HSD17B7	ko00100	K13373	1.1.1.270	Oxidoreductases	R074495	17beta-estradiol 17-dehydrogenase / 3beta-hydroxysteroid 3-dehydrogenase	100440_c1_g1; 74062_c0_g1	122943861
EBP	ko00100	K01824	5.3.3.5	Isomerases	R04804	Cholestenol Delta-isomerase	95273_c4_g1	122919324
DHCR24	ko00100	K09828	1.3.1.72	Oxidoreductases	R07498	Delta24-sterol reductase	94163_c2_g3; 80208_c0_g1	122943331
SC5DL	ko00100	K00227	1.14.19.20	Oxidoreductases	R07215	Delta7-sterol 5-desaturase	95499_c4_g8	122926803
DHCR7	ko00100	K00213	1.14.13.78	Oxidoreductases	R01451 R01456	7-dehydrocholesterol reductase	100322_c1_g1	122920404
SOAT	ko00100	K00637	2.3.1.26	Transferases	R01461	sterol O-acyltransferase	109197_c0_g1; 95001_c0_g1	122931422; 122931423; 122932620; 122943182

Note: Original local transcript identifiers are retained for traceability. NCBI Gene IDs were assigned based on corresponding KEGG entries and the current *Bufo gargarizans* genome annotation.

Table S5 Bioinformatics of *Bg-3βHSDs* from *B. bufo gargarizans*

Gene name	GenBank accession number	CDS (bp)	Amino acids	MW (kDa)	instability indices	IP	TMD	Subcellular localization
Bg3βHSD1	MG517527	1134	377	42.8	42.79	8.54	0	E
Bg3βHSD2	MT328812	1104	367	41.8	36.48	8.72	1	C
Bg3βHSD3	MT328813	861	286	33.1	45.14	9.15	0	C
Bg3βHSD4	MT328814	1038	345	38.6	42.45	8.70	1	C
Bg3βHSD5	MT328815	1176	391	43.6	40.10	9.35	2	E.R.
Bg3βHSD6	MT328816	1122	373	42.1	34.46	8.85	0	M
Bg3βHSD7	MT328817	1323	440	50.0	46.15	7.98	2	P
Bg-HSD3B7	XM_044303756.1	1107	368	41.5	43.11	7.27	0	C

Note: CDS, coding DNA sequence; MW, molecular weight; IP, isoelectric point; E, extracellular; C, Cytoplasmic; E.R, Endoplasmic reticulum and mitochondrial membrane-bound protein; M, Mitochondrial matrix; P, Integral membrane protein; TMD, transmembrane domain

Table S6 Identities at amino acid level between Bg-3βHSDs in this study

Identity (%)	**Bg3βHSD1**	**Bg3βHSD2**	Bg3βHSD3	Bg3βHSD4	Bg3βHSD5	Bg3βHSD6	**Bg3βHSD7**	BgHSD3B7
**Bg3βHSD1**	-							
**Bg3βHSD2**	**34.06**	-						
Bg3βHSD3	18.25	15.61	-					
Bg3βHSD4	27.57	28.57	17.83	-				
Bg3βHSD5	27.61	27.22	15.41	32.35	-			
Bg3βHSD6	51.48	30.68	16.85	28.06	30.77	-		
**Bg3βHSD7**	**28.85**	**27.92**	12.69	31.29	40.73	26.63	-	
Bg-HSD3B7	35.97	40.38	13.91	30.29	30.29	34.25	26.99	-

Note: Active 3βHSDs identified in this study are highlighted in bold.

Table S7 Information of reported 3βHSDs used in the study for functional clustering

Functional clustering	Entry name	UniProt accession number	GenBank accession number	Entry function	Tissue site
C-19/C-21 3βHSD	3BHS1_MOUSE	P24815	M58567	pregnenolone+NAD>pregn-5-ene-3,20-dione 3β-hydroxyandrost-5-en-17-one+NAD>androst-5-ene-3,17-dione androst-5-en-3β,17β-diol+NAD>17β-hydroxy-androst-5-en-3-one 5α-androstane-3β,17β-diol+NADP>17β-hydroxy-5α-androstan-3-one 5α-androstane-3β,17β-diol+NAD>17β-hydroxy-5α-androstan-3-one 3β-hydroxy-5α-androstan-17-one+NADP>5α-androstan-3,17-dione	testis, adrenal glands, kidney, neonatal liver
C-19/C-21 3βHSD	3BHS2_MOUSE	P26149	M75886	pregnenolone+NAD>pregn-5-ene-3,20-dione 3β-hydroxyandrost-5-en-17-one+NAD>androst-5-ene-3,17-dione	liver, kidney
C-19/C-21 3βHSD	3BHS3_MOUSE	P26150	M77015	pregnenolone+NAD>pregn-5-ene-3,20-dione 3β-hydroxyandrost-5-en-17-one+NAD>androst-5-ene-3,17-dione	liver, kidney
C-19/C-21 3βHSD	3BHS4_MOUSE	Q61767	L16919	5α-androstane-3β,17β-diol+NADP>17β-hydroxy-5α-androstan-3-one	liver
C-19/C-21 3βHSD	3BHS6_MOUSE	O35469	AF031170	pregnenolone+NAD>pregn-5-ene-3,20-dione 3β-hydroxyandrost-5-en-17-one+NAD>androst-5-ene-3,17-dione	trophoblast, testis
C-27 3βHSD	3BHS7_MOUSE	Q9EQC1	AF277718	7α-hydroxycholesterol+NAD>7α-hydroxycholest-4-en-3-one 7α,25-dihydroxycholesterol+NAD>7α,25-dihydroxy-4-cholesten-3-one (25R)-cholest-5-en-3β,7α,26-triol+NAD>(25R)-7α,26-dihydroxycholest-4-en-3-one (24S)-7α-dihydroxycholesterol+NAD>(24S)-7α,24-dihydroxycholest-4-en-3-one	

Functional clustering	Entry name	UniProt accession number	GenBank accession number	Entry function	Tissue site
C-19/C-21 3βHSD	3BHS1_RAT	P22071	M38178	pregnenolone+NAD>pregn-5-ene-3,20-dione 3β-hydroxyandrost-5-en-17-one+NAD>androst-5-ene-3,17-dione androst-5-en-3β,17β-diol+NAD>17β-hydroxy-androst-5-en-3-one 5α-androstane-3β,17β-diol+NADP>17β-hydroxy-5α-androstan-3-one 5α-androstane-3β,17β-diol+NAD>17β-hydroxy-5α-androstan-3-one 3β-hydroxy-5α-androstan-17-one+NADP>5α-androstan-3,17-dione	adrenal glands, ovary, testis, embryo, kidney, adipose tissue, uterus
C-19/C-21 3βHSD	3BHS2_RAT	P22072	M38179	pregnenolone+NAD>pregn-5-ene-3,20-dione 3β-hydroxyandrost-5-en-17-one+NAD>androst-5-ene-3,17-dione androst-5-en-3β,17β-diol+NAD>17β-hydroxy-androst-5-en-3-one 5α-androstane-3β,17β-diol+NADP>17β-hydroxy-5α-androstan-3-one 5α-androstane-3β,17β-diol+NAD>17β-hydroxy-5α-androstan-3-one 3β-hydroxy-5α-androstan-17-one+NADP>5α-androstan-3,17-dione	adrenal glands, ovary, testis, embryo, kidney, adipose tissue, uterus
C-19/C-21 3βHSD	3BHS4_RAT	Q62878	L17138	pregnenolone+NAD>pregn-5-ene-3,20-dione 3β-hydroxyandrost-5-en-17-one+NAD>androst-5-ene-3,17-dione	skin, adrenal glands, ovary, embryo
C-19/C-21 3βHSD	3BHS5_RAT	P27364	M67465	5α-androstane-3β,17β-diol+NADP>17β-hydroxy-5α-androstan-3-one 3β-hydroxy-5α-androstan-17-one+NADP>5α-androstan-3,17-dione	liver
C-27 3βHSD	3BHS7_RAT	O35048	AB000199	7α-hydroxycholesterol+NAD>7α-hydroxycholest-4-en-3-one 7α,25-dihydroxycholesterol+NAD>7α,25-dihydroxy-4-cholesten-3-one (25R)-cholest-5-en-3β,7α,26-triol+NAD>(25R)-7α,26-dihydroxycholest-4-en-3-one (24S)-7α-dihydroxycholesterol+NAD>(24S)-7α,24-dihydroxycholest-4-en-3-one	liver

Functional clustering	Entry name	UniProt accession number	GenBank accession number	Entry function	Tissue site
C-19/C-21 3βHSD	3BHS1_MESAU	Q60555	L38709	pregnenolone+NAD>pregn-5-ene-3,20-dione 3β-hydroxyandrost-5-en-17-one+NAD>androst-5-ene-3,17-dione androst-5-en-3β,17β-diol+NAD>17β-hydroxy-androst-5-en-3-one 5α-androstane-3β,17β-diol+NADP>17β-hydroxy-5α-androstan-3-one 5α-androstane-3β,17β-diol+NAD>17β-hydroxy-5α-androstan-3-one 3β-hydroxy-5α-androstan-17-one+NADP>5α-androstan-3,17-dione	adrenal glands
C-19/C-21 3βHSD	3BHS2_MESAU	Q64421	L38710	pregnenolone+NAD>pregn-5-ene-3,20-dione 3β-hydroxyandrost-5-en-17-one+NAD>androst-5-ene-3,17-dione	kidney, liver
C-19/C-21 3βHSD	3BHS3_MESAU	O35296	AF017636	5α-androstane-3β,17β-diol+NADP>17β-hydroxy-5α-androstan-3-one	liver
C-27 3βHSD	3BHS7_HUMAN	Q9H2F3	AF277719	7α-hydroxycholesterol+NAD>7α-hydroxycholest-4-en-3-one 7α,25-dihydroxycholesterol+NAD>7α,25-dihydroxy-4-cholesten-3-one (25R)-cholest-5-en-3β,7α,26-triol+NAD>(25R)-7α,26-dihydroxycholest-4-en-3-one (24S)-7α-dihydroxycholesterol+NAD>(24S)-7α,24-dihydroxycholest-4-en-3-one	liver
C-19/C-21 3βHSD	3BHS1_HUMAN	P14060	M27137	pregnenolone+NAD>pregn-5-ene-3,20-dione 3β-hydroxyandrost-5-en-17-one+NAD>androst-5-ene-3,17-dione androst-5-en-3β,17β-diol+NAD>17β-hydroxy-androst-5-en-3-one 5α-androstane-3β,17β-diol+NADP>17β-hydroxy-5α-androstan-3-one 5α-androstane-3β,17β-diol+NAD>17β-hydroxy-5α-androstan-3-one 3β-hydroxy-5α-androstan-17-one+NADP>5α-androstan-3,17-dione	adrenal glands, testis, ovary, placenta, prostate, breast, liver, skin

Functional clustering	Entry name	UniProt accession number	GenBank accession number	Entry function	Tissue site
C-19/C-21 3βHSD	3BHS2_HUMAN	P26439	M77144	pregnenolone+NAD>pregn-5-ene-3,20-dione 3β-hydroxyandrost-5-en-17-one+NAD>androst-5-ene-3,17-dione	adrenal glands, gonad
C-19/C-21 3βHSD	3BHS1_MACMU	P27365	M67467	pregnenolone+NAD>pregn-5-ene-3,20-dione 3β-hydroxyandrost-5-en-17-one+NAD>androst-5-ene-3,17-dione androst-5-en-3β,17β-diol+NAD>17β-hydroxy-androst-5-en-3-one 5α-androstane-3β,17β-diol+NADP>17β-hydroxy-5α-androstan-3-one 5α-androstane-3β,17β-diol+NAD>17β-hydroxy-5α-androstan-3-one 3β-hydroxy-5α-androstan-17-one+NADP>5α-androstan-3,17-dione	ovary, testis, adrenal glands, liver, kidney, epididymis
C-27 3βHSD	HSD3B_CAEEL	Q9XWF0	BX284601	cholesterol+NAD>4-Cholesten-3-one	
C-19/C-21 3βHSD	3BHS_BOVIN	P14893	X17614		ovary
C-19/C-21 3βHSD	3BHS_PIG	Q9N119	AF232699		adipose tissue
C-19/C-21 3βHSD	3BHS_HORSE	O46516	AF031665	pregnenolone+NAD>pregn-5-ene-3,20-dione 3β-hydroxyandrost-5-en-17-one+NAD>androst-5-ene-3,17-dione	ovary
C-19/C-21	3BHS_CANLF	Q5IFP1	AY739720	pregnenolone+NAD>pregn-5-ene-3,20-dione 3β-hydroxyandrost-5-en-17-one+NAD>androst-5-ene-3,17-dione	ovary
C-19/C-21 3βHSD	3BHS2_HUMAN	P26439	M77144	pregnenolone+NAD>pregn-5-ene-3,20-dione 3β-hydroxyandrost-5-en-17-one+NAD>androst-5-ene-3,17-dione	adrenal glands, gonad

Functional clustering	Entry name	UniProt accession number	GenBank accession number	Entry function	Tissue site
C-19/C-21 3βHSD	3BHS1_MACMU	P27365	M67467	pregnenolone+NAD>pregn-5-ene-3,20-dione<br>3β-hydroxyandrost-5-en-17-one+NAD>androst-5-ene-3,17-dione<br>androst-5-en-3β,17β-diol+NAD>17β-hydroxy-androst-5-en-3-one<br>5α-androstane-3β,17β-diol+NADP>17β-hydroxy-5α-androstan-3-one<br>5α-androstane-3β,17β-diol+NAD>17β-hydroxy-5α-androstan-3-one<br>3β-hydroxy-5α-androstan-17-one+NADP>5α-androstan-3,17-dione	ovary, testis, adrenal glands, liver, kidney, epididymis
C-27 3βHSD	HSD3B_CAEEL	Q9XWF0	BX284601	cholesterol+NAD>4-Cholesten-3-one	
C-19/C-21 3βHSD	3BHS_BOVIN	P14893	X17614		ovary
C-19/C-21 3βHSD	3BHS_PIG	Q9N119	AF232699		adipose tissue
C-19/C-21 3βHSD	3BHS_HORSE	O46516	AF031665	pregnenolone+NAD>pregn-5-ene-3,20-dione<br>3β-hydroxyandrost-5-en-17-one+NAD>androst-5-ene-3,17-dione	ovary
C-19/C-21	3BHS_CANLF	Q5IFP1	AY739720	pregnenolone+NAD>pregn-5-ene-3,20-dione<br>3β-hydroxyandrost-5-en-17-one+NAD>androst-5-ene-3,17-dione	ovary

Table S8 Correlation analysis between gene expression and bufadienolides in the control samples

Items	*Bg3βHSD1*	*Bg3βHSD2*	*Bg3βHSD3*	*Bg3βHSD4*	*Bg3βHSD5*	*Bg3βHSD7*	Resibufogenin	Cino bufagin	Bufalin	Telocino bufagin
Bg3βHSD1	1.000									
Bg3βHSD2	-0.328	1.000								
Bg3βHSD3	-0.398	0.703	1.000							
Bg3βHSD4	-0.114	-0.556	-0.865**	1.000						
Bg3βHSD5	0.319	0.239	-0.510	0.409	1.000					
Bg3βHSD7	1.000**	-0.321	-0.402	-0.110	0.331	1.000				
Resibufogenin	0.548	-0.581	0.021	-0.351	-0.610	0.537	1.000			
Cinobufagin	0.583	-0.570	0.012	-0.359	-0.578	0.573	0.999**	1.000		
Bufalin	0.555	-0.567	0.032	-0.366	-0.606	0.544	1.000**	0.999**	1.000	
Telocinobufagin	0.056	-0.478	0.284	-0.372	-0.922**	0.043	0.865**	0.843*	0.862**	1.000

Note: * and ** indicate significant correlations at $P < 0.05$ and $P < 0.01$, respectively.

Table S9 Correlation analysis between gene expression and bufadienolide levels in $PbSO_4$ treated samples.

Items	*Bg3βHSD1*	*Bg3βHSD2*	*Bg3βHSD3*	*Bg3βHSD4*	*Bg3βHSD5*	*Bg3βHSD6*	*Bg3βHSD7*	Resibufogenin	Cinobufagin	Bufalin	Telocinobufagin
Bg3βHSD1	1.000										
Bg3βHSD2	0.886**	1.000									
Bg3βHSD3	0.702	0.912**	1.000								
Bg3βHSD4	-0.813	-0.546	-0.487	1.000							
Bg3βHSD5	0.181	-0.096	-0.497	0.005	1.000						
Bg3βHSD6	0.882**	1.000**	0.914**	-0.541	-0.103	1.000					
Bg3βHSD7	0.053	-0.415	-0.598	-0.404	0.590	-0.423	1.000				
Resibufogenin	0.657	0.233	-0.025	-0.797*	0.573	0.224	0.788*	1.000			
Cinobufagin	0.127	-0.339	-0.585	-0.384	0.718	-0.347	0.984**	0.825*	1.000		
Bufalin	0.100	-0.369	-0.589	-0.397	0.667	-0.377	0.995**	0.815*	0.997**	1.000	
Telocinobufagin	0.167	-0.304	-0.543	-0.435	0.692	-0.312	0.986**	0.850**	0.998**	0.997**	1.000

Note: * and ** indicate significant correlations at $P < 0.05$ and $P < 0.01$, respectively.

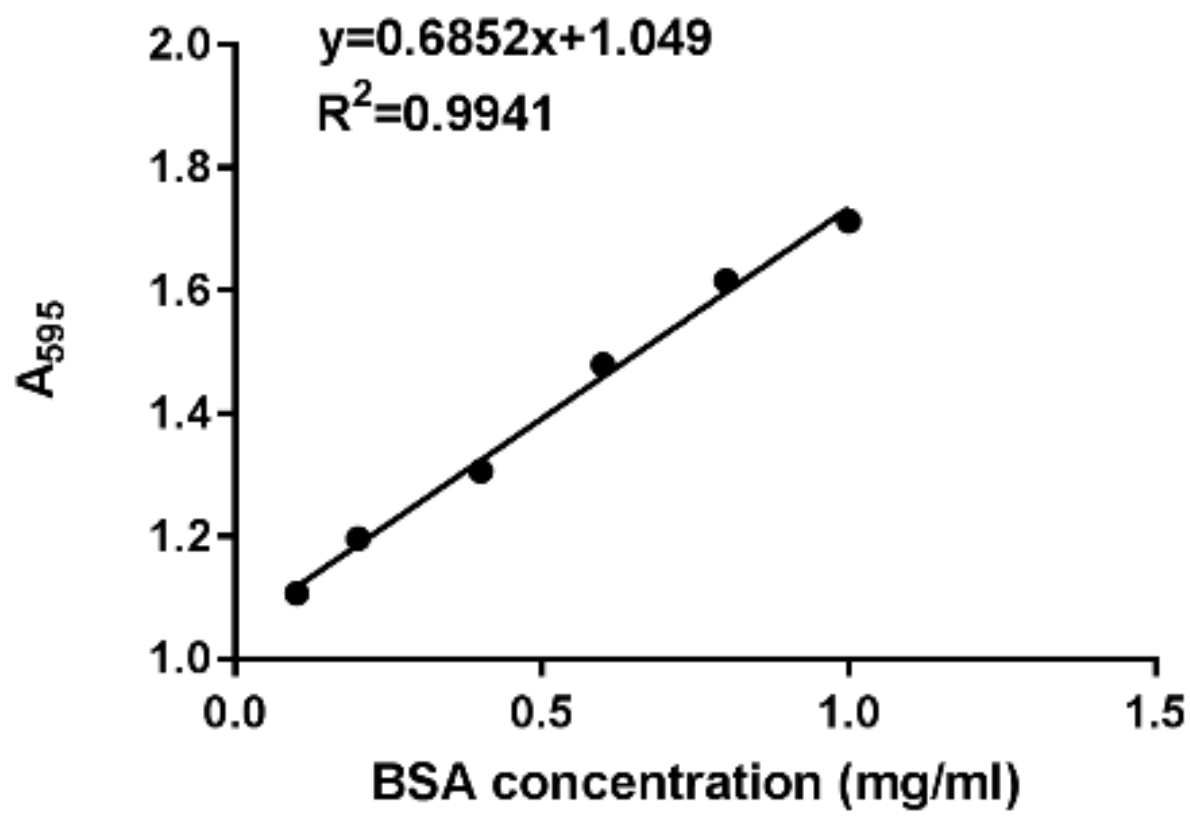

Fig. S1 Standard curve of bovine serum albumin (BSA)

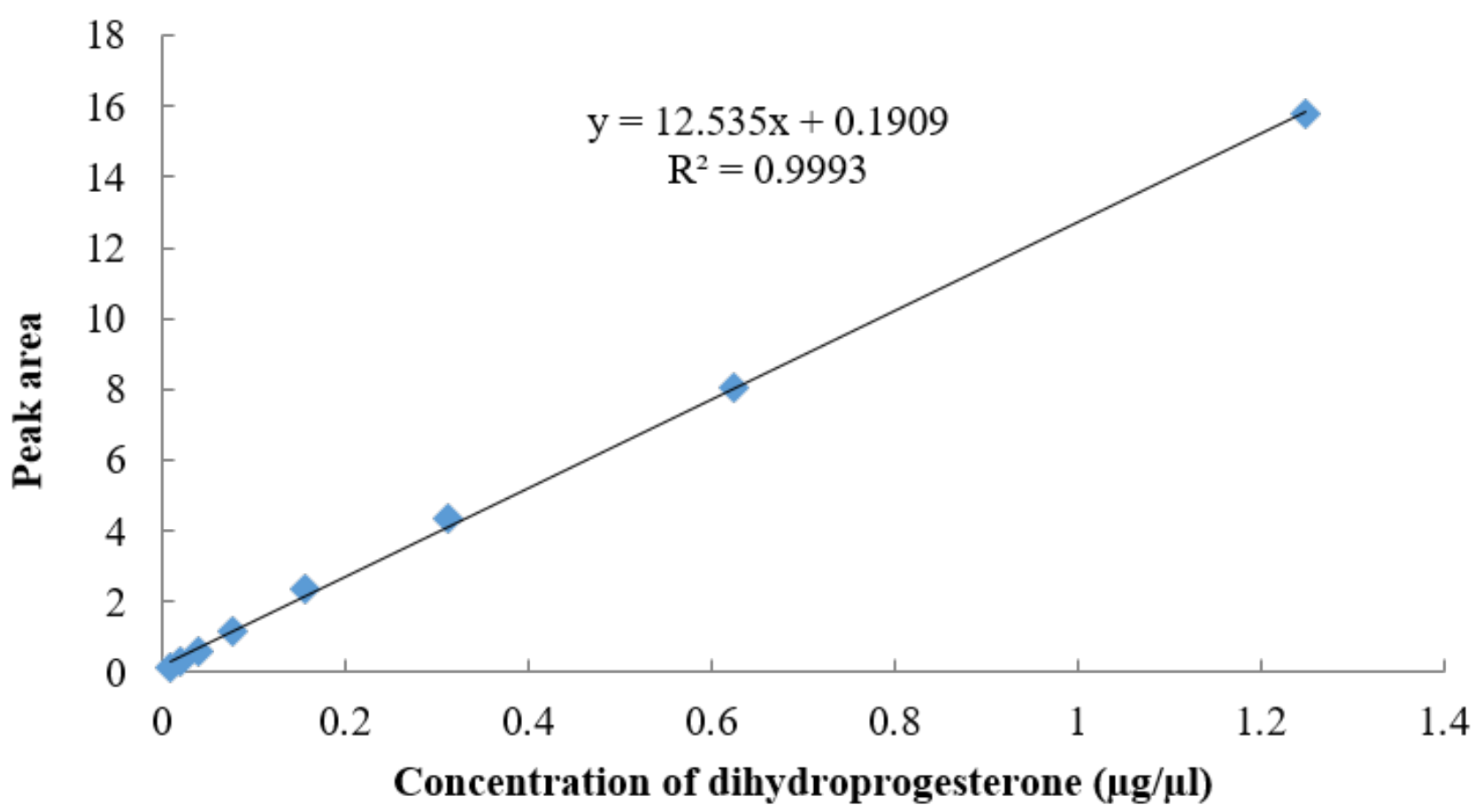

Fig. S2 Standard reference curve of dihydroprogesterone

A

Type	Resource
Total transcripts number	238,690
Total unigenes number	183,776
Total sequence base	189,422,945
Largest	29,933
Smallest	201
Average length	793.59
N50	1151
GC percent	45.38

C

Type	Transcript number(percent)	Unigene number(percent)
NR	66414(0.2782)	41768(0.2273)
Swiss-Prot	55763(0.2336)	35107(0.191)
Pfam	45844(0.1921)	29604(0.1611)
COG	17235(0.0722)	10842(0.059)
GO	22832(0.0957)	13329(0.0725)
KEGG	40360(0.1691)	25687(0.1398)
Total_annotation	73209(0.3067)	47758(0.2599)
Total	238690(1)	183776(1)

B

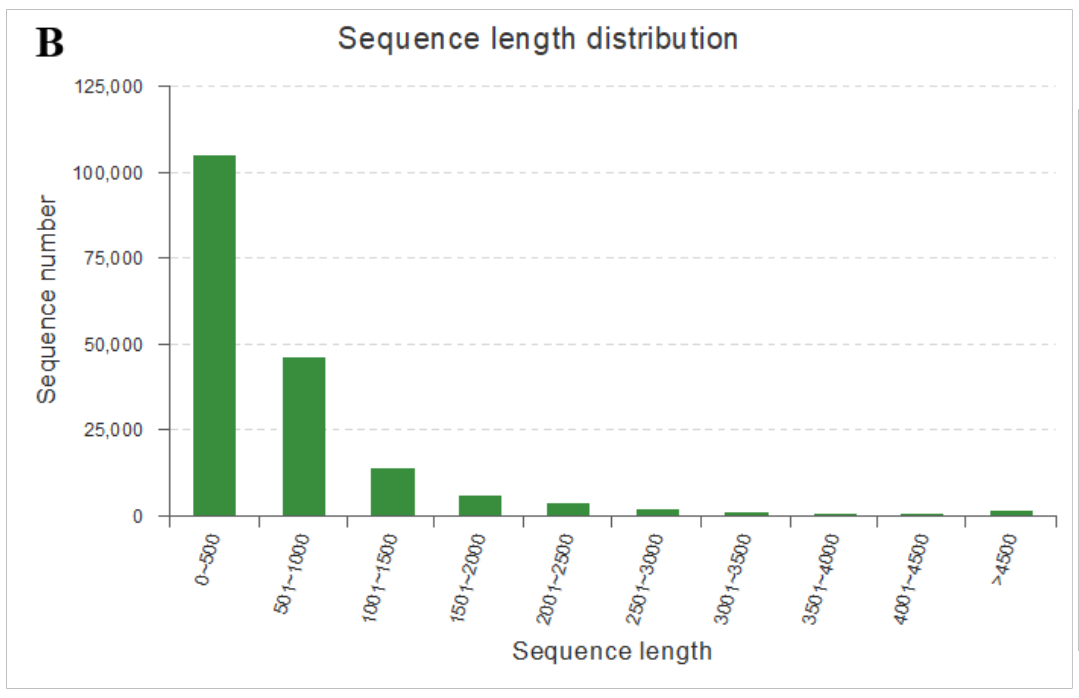

D

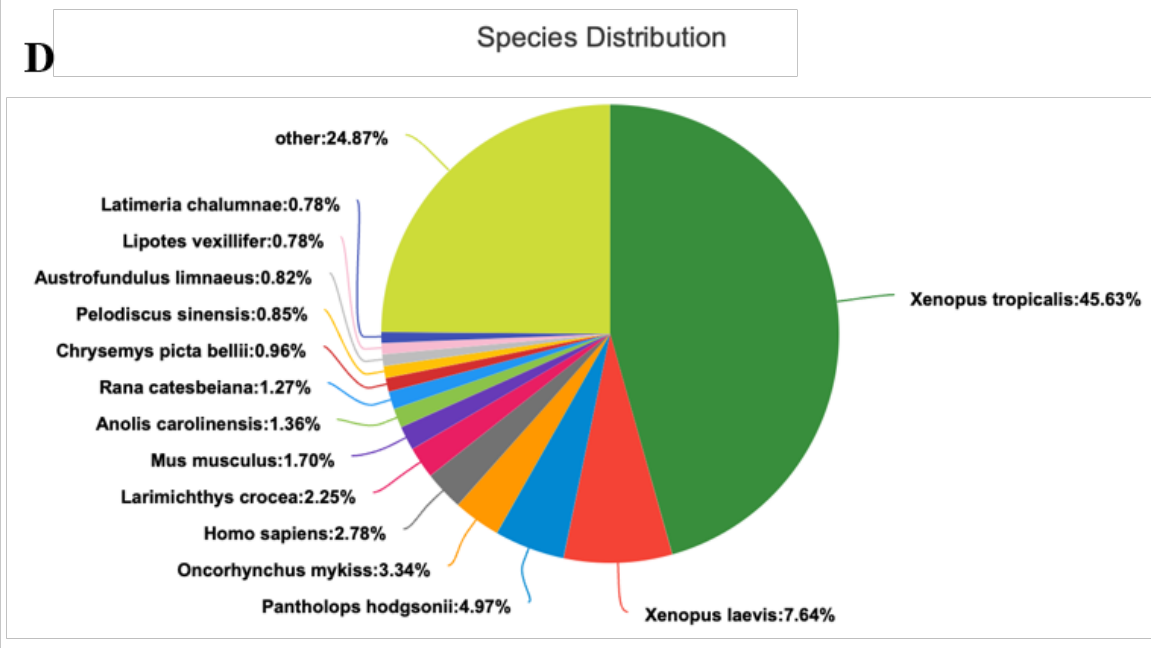

Fig. S3 Results of de novo transcriptome assembly of *B. bufo gargarizans*.

(A) Assembly statistics of the assembled transcripts.

(B) Length distribution of unigenes assembled using Trinity.

(C) Number of transcripts and unigenes annotated in different databases.

(D) Species distribution of homologous sequences identified in the NR database.

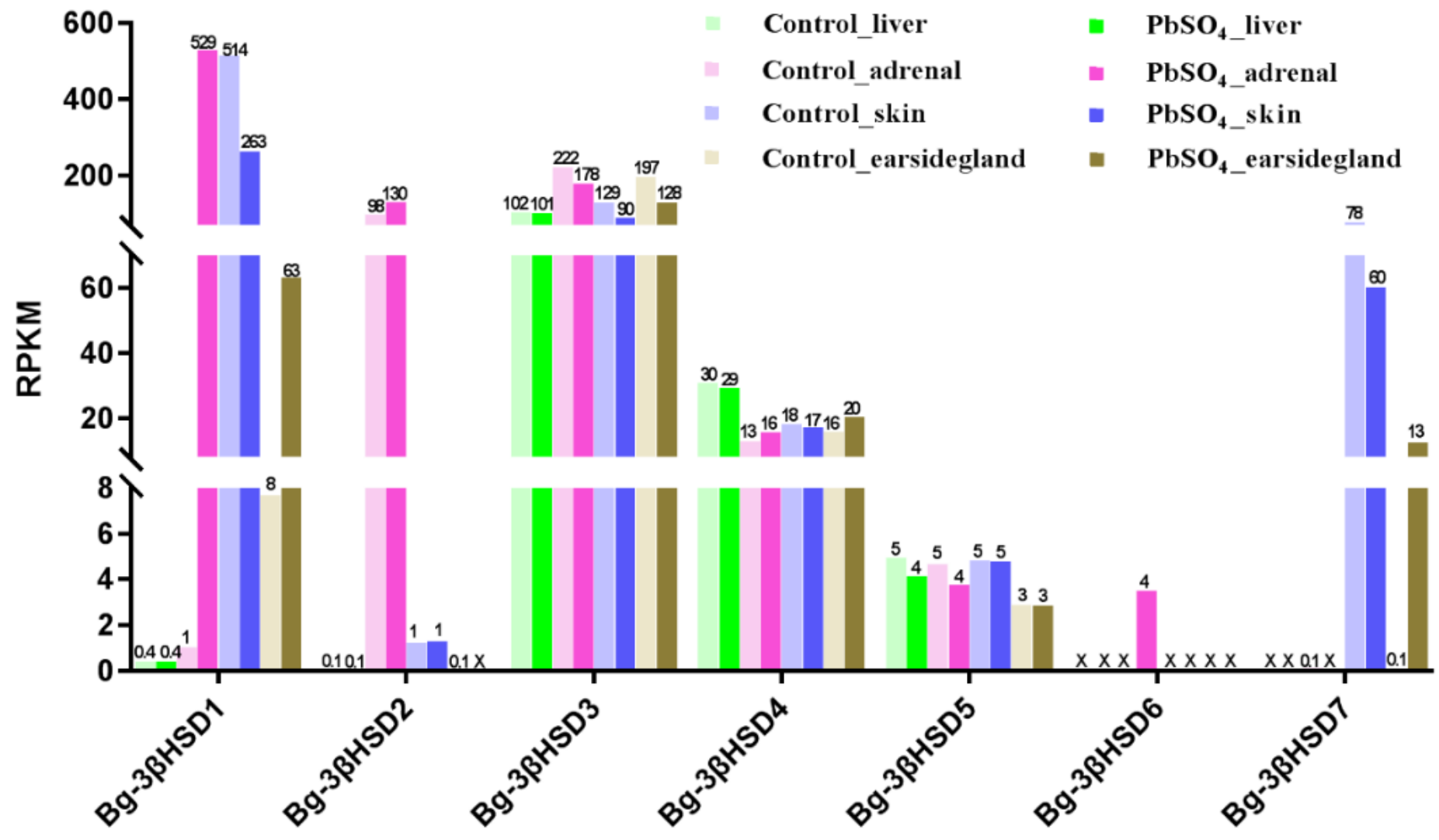

Fig. S4 Expression patterns of Bg-3βHSD genes in control and $PbSO_4$-treated *B. bufo gargarizans* samples.

The numbers above the columns represent the Reads Per Kilobase of transcript per Million mapped reads (RPKM) values obtained from the *B. bufo gargarizans* transcriptome dataset generated in this study.

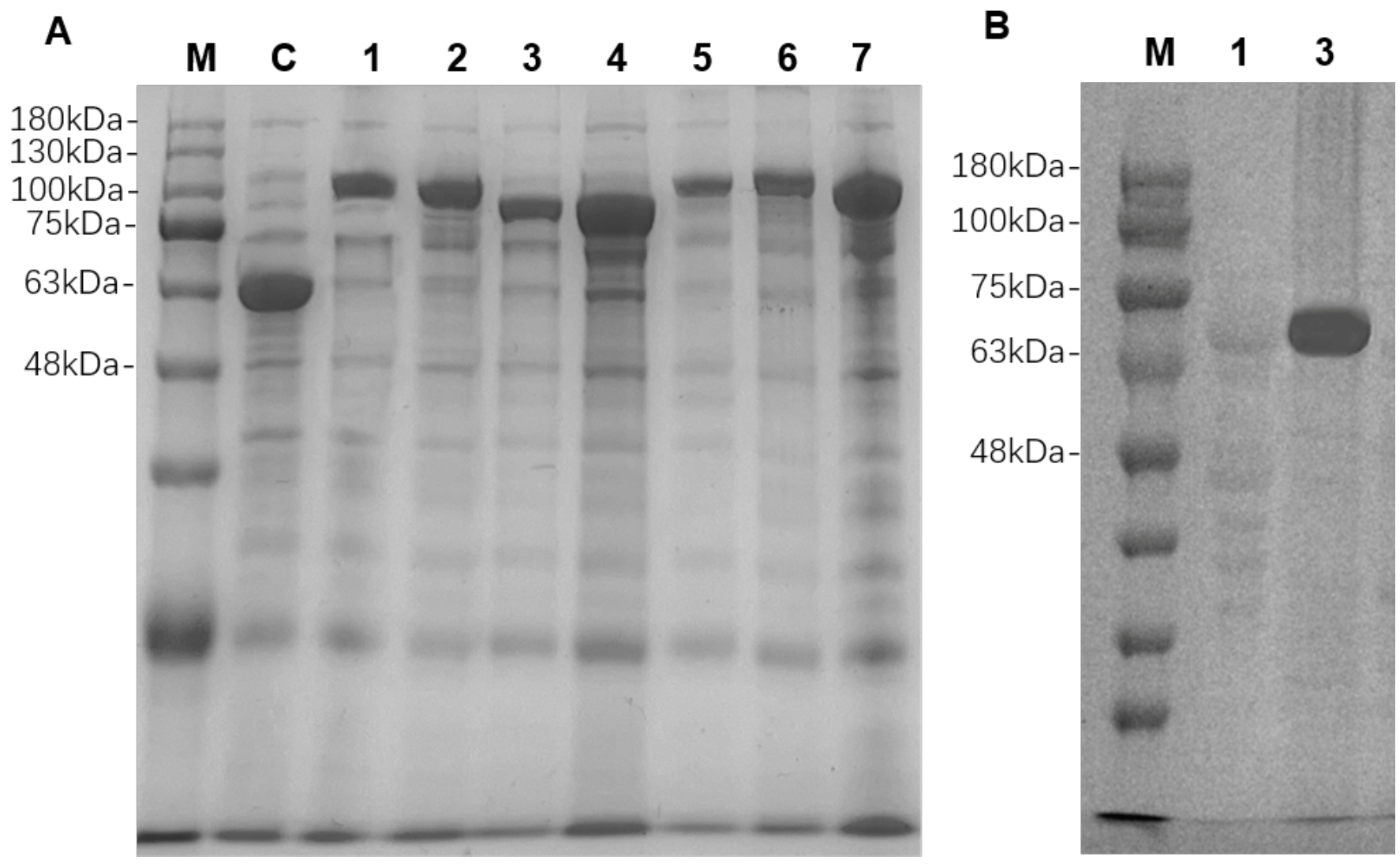

Fig. S5 SDS-PAGE analysis of recombinant Bg-3βHSD proteins and purification of recombinant Bg-3βHSD2.

(A) SDS-PAGE analysis of soluble protein extracts from *E. coli* strains expressing recombinant Bg-3βHSDs. M, protein molecular weight marker; C, soluble protein extract from E. coli cells harboring the empty pCold vector; lanes 1-5, soluble protein extracts from E. coli cells expressing Bg-3βHSD1-5, respectively; lane 6, soluble protein extract from *E. coli* cells expressing Bg-3βHSD7; lane 7, soluble protein extract from *E. coli* cells expressing Bg-HSD3B7. ImageJ densitometric analysis of SDS-PAGE band intensities indicated that recombinant Bg-3βHSD1, Bg-3βHSD2, Bg-3βHSD3, Bg-3βHSD4, Bg-3βHSD5, Bg-3βHSD7, and Bg-HSD3B7 represented 9.90%，10.43%，13.22%，39.26%，11.24%, 10.07%, and 21.52% of the total soluble protein, respectively.

(B) SDS-PAGE analysis of purification of recombinant Bg-3βHSD2 by Ni-NTA affinity chromatography. M, protein molecular weight marker; 1, elution fraction collected during purification; 2, purified recombinant Bg-3βHSD2 protein.

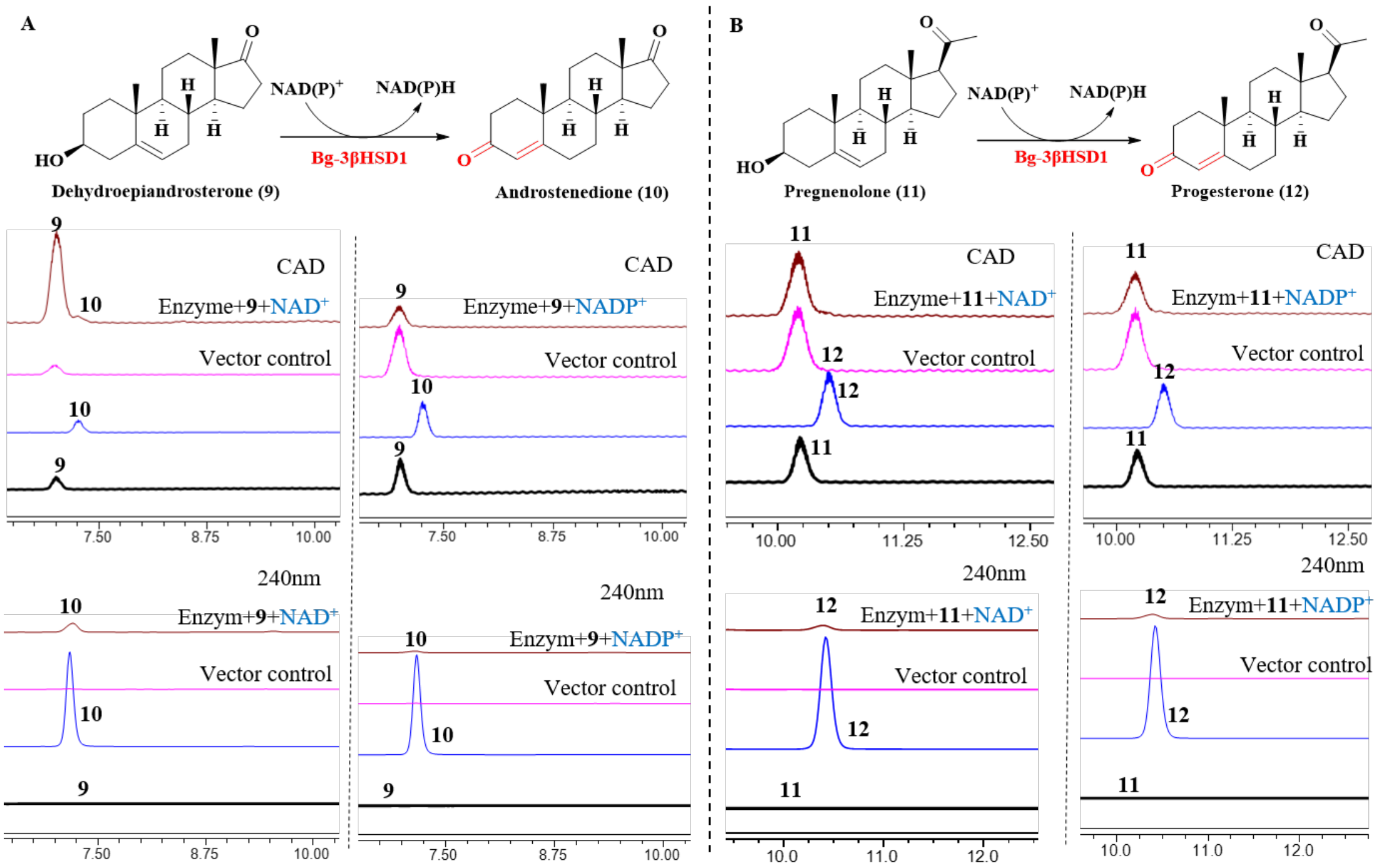

Fig. S6 Representative HPLC chromatograms showing the oxidative activity of recombinant Bg-3βHSD1 enzyme toward dehydroepiandrosterone (9) and pregnenolone (11).

Oxidative conversion of dehydroepiandrosterone (**9**) (A) and pregnenolone (**11**) (B) catalyzed by recombinant Bg-3βHSD1 enzyme, detected by CAD and DAD at 240 nm. Oxidation reactions were performed using NAD^+ or $NADP^+$ as cofactors. In each chromatogram, traces are arranged from bottom to top as follows: authentic substrate standard, authentic product standard, reaction mixture containing crude extracts from *E. coli* cells harboring the empty pCold vector (Vector control), and reaction mixture containing crude extracts from *E. coli* cells expressing recombinant Bg-3βHSD1 together with the indicated substrate and cofactor. Compound numbers correspond to those assigned in Table 1 and are shown in the reaction schemes above the corresponding chromatograms.

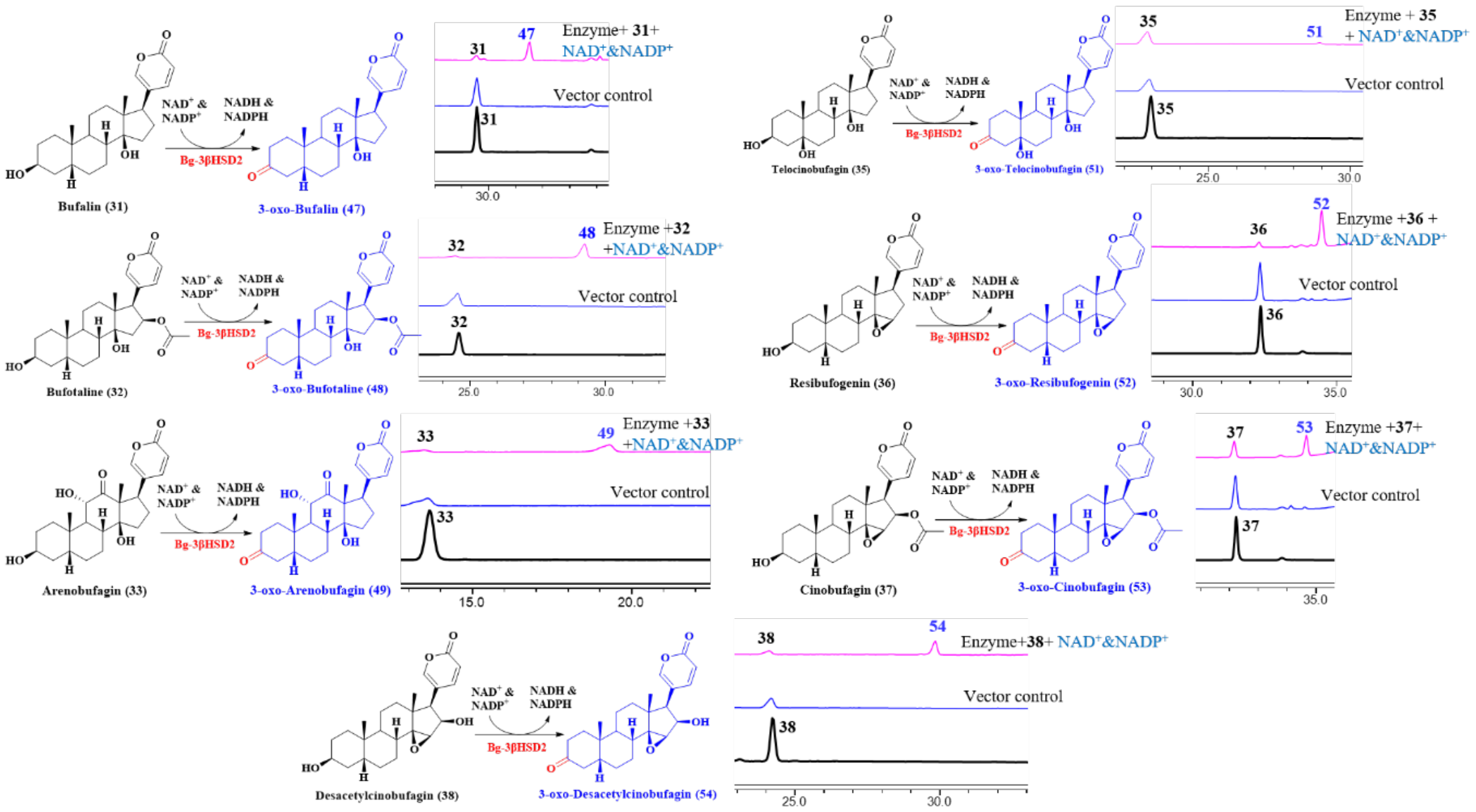

Fig. S7 Representative HPLC chromatograms showing the oxidative activity of recombinant Bg-3βHSD2 enzyme toward bufadienolide substrates.

Oxidative conversion of bufadienolides **31**, **32**, **33** (up-left panel), **35**, **36**, **37** (up-right panel), and **38** (bottom panel) catalyzed by recombinant Bg-3βHSD2 enzyme, detected by CAD. Oxidation reactions were performed using a mixture of NAD^+ and $NADP^+$ as cofactors. Authentic standards were available only for the substrates. In each chromatogram, traces are arranged from bottom to top as follows: authentic substrate standard, reaction mixture containing crude extracts from *E. coli* cells harboring the empty pCold vector (Vector control), and reaction mixture containing crude extracts from *E. coli* cells expressing recombinant Bg-3βHSD2 together with the indicated substrate and cofactors. Compound numbers correspond to those assigned in Table 1 and are shown in the reaction schemes adjacent to the corresponding chromatograms.

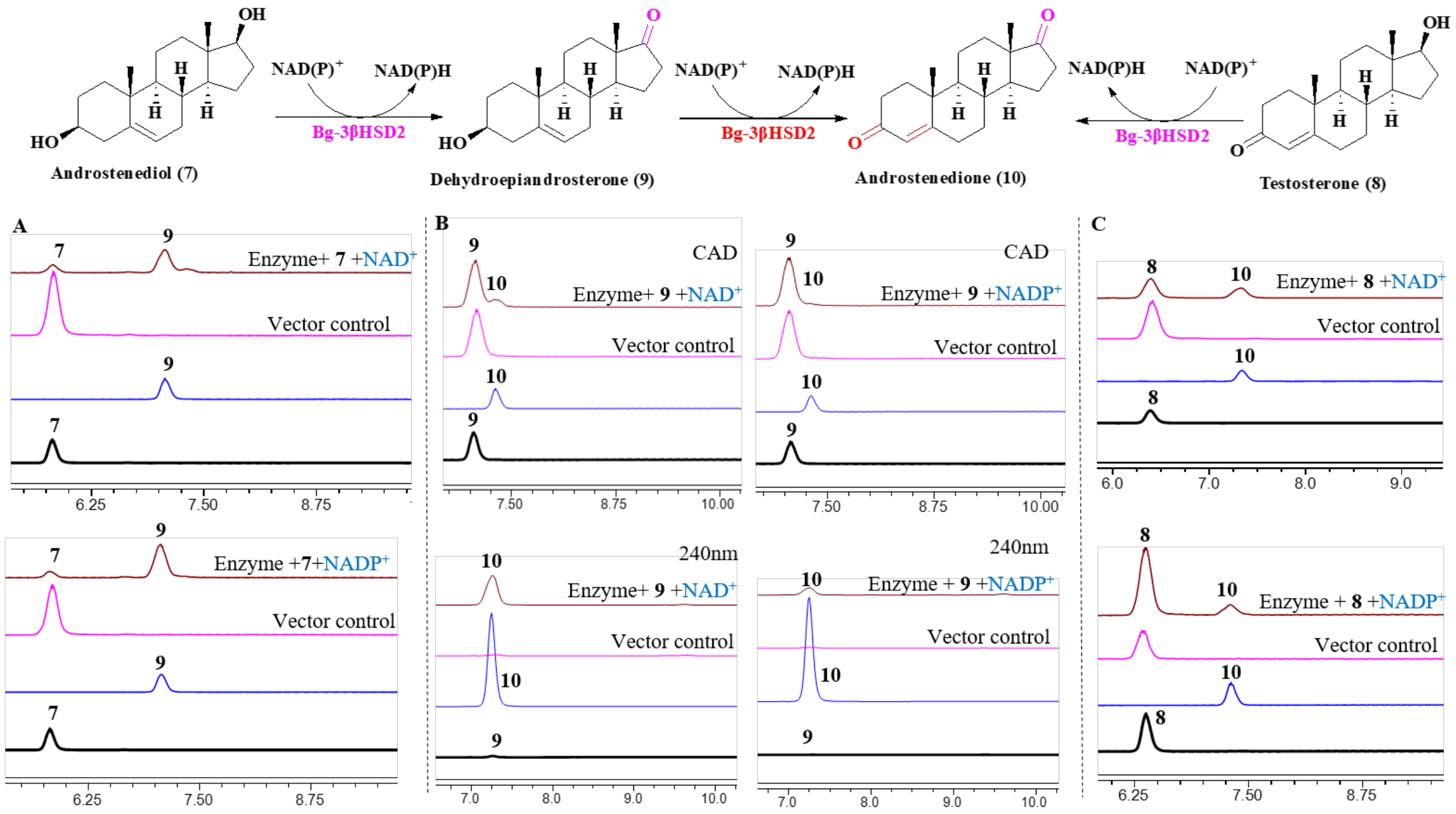

Fig. S8 Representative HPLC chromatograms showing the oxidative activity of recombinant Bg-3βHSD2 enzyme toward hormone substrates.

Oxidative conversion of androstenediol (**7**) (A), dehydroepiandrosterone (**9**) (B), and testosterone (**8**) (C) catalyzed by recombinant Bg-3βHSD2 enzyme, detected by CAD and DAD at 240 nm. Oxidation reactions were performed using NAD^+ or $NADP^+$ as cofactors. In each chromatogram, traces are arranged from bottom to top as follows: authentic substrate standard, authentic product standard, reaction mixture containing crude extracts from *E. coli* cells harboring the empty pCold vector (Vector control), and reaction mixture containing crude extracts from *E. coli* cells expressing recombinant Bg-3βHSD2 together with the indicated substrate and cofactor. Compound numbers correspond to those assigned in Table 1 and are shown in the reaction schemes above the corresponding chromatograms.

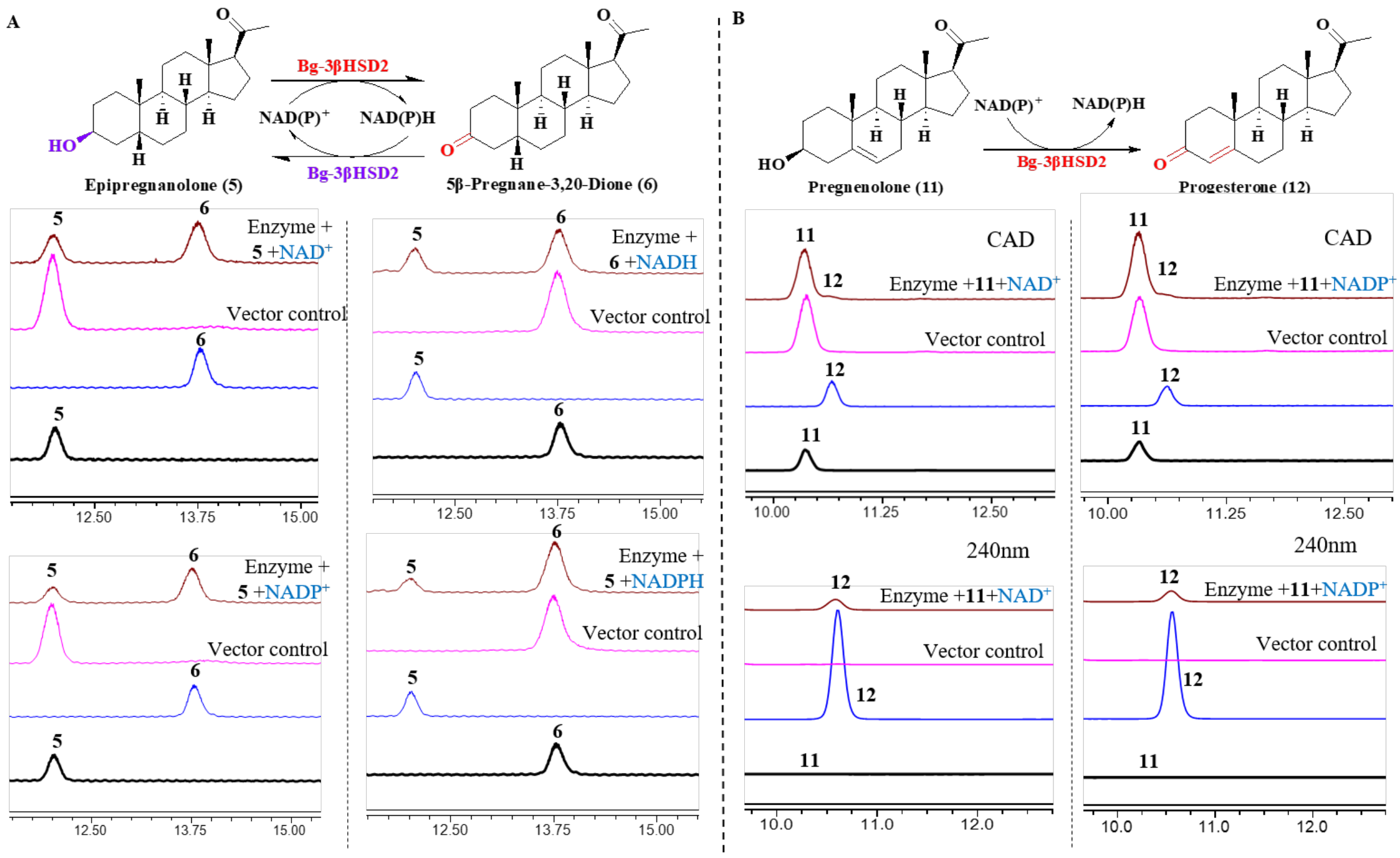

Fig. S9 Representative HPLC chromatograms showing the redox activity of recombinant Bg-3βHSD2 enzyme toward hormone substrates.

Bidirectional redox conversion between epipregnanolone (**5**) and 5β-pregnane-3,20-dione (**6**) (A), and oxidative conversion of pregnenolone (**11**) (B) catalyzed by recombinant Bg-3βHSD2 enzyme. Chromatograms were detected by CAD, except for pregnenolone (**11**), which was detected by both CAD (upper panel) and DAD at 240 nm (lower panel). Oxidation reactions were performed using NAD^+ or $NADP^+$ as cofactors, whereas reduction reactions were performed using NADH or NADPH. In each chromatogram, traces are arranged from bottom to top as follows: authentic substrate standard, authentic product standard, reaction mixture containing crude extracts from *E. coli* cells harboring the empty pCold vector (Vector control), and reaction mixture containing crude extracts from *E. coli* cells expressing recombinant Bg-3βHSD2 together with the indicated substrate and cofactor. Compound numbers correspond to those assigned in Table 1 and are shown in the reaction schemes above the corresponding chromatograms.

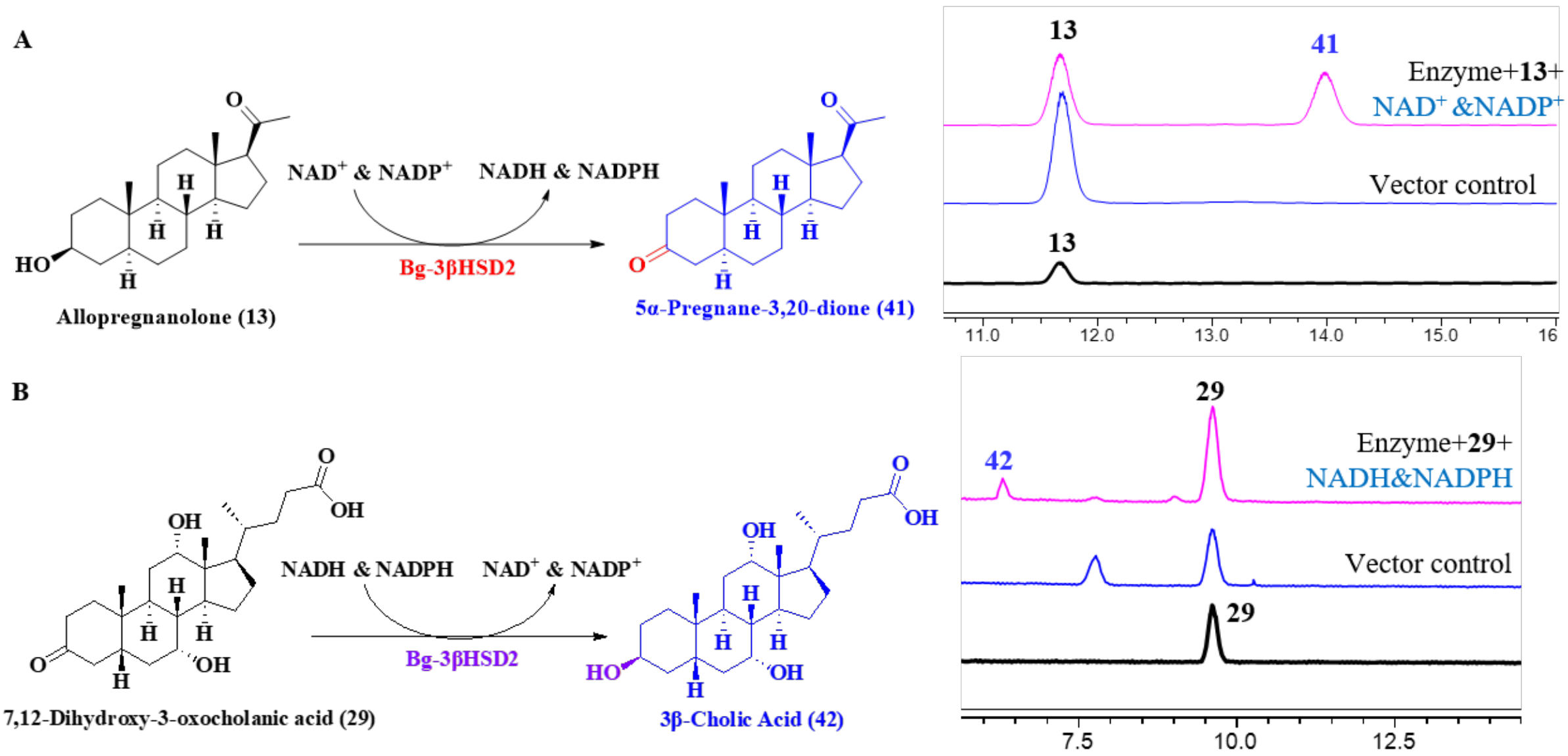

Fig. S10 Representative HPLC chromatograms showing the oxidative activity of recombinant Bg-3βHSD2 enzyme toward steroid substrates.

Oxidative conversion of allopregnanolone (**13**) (A) and 7,12-dihydroxy-3-oxocholanic acid (**29**) (B) catalyzed by recombinant Bg-3βHSD2 enzyme, detected by CAD. Oxidation reactions were performed using NAD^+ or $NADP^+$ as cofactors. In each chromatogram, traces are arranged from bottom to top as follows: authentic substrate standard, reaction mixture containing crude extracts from *E. coli* cells harboring the empty pCold vector (Vector control), and reaction mixture containing crude extracts from *E. coli* cells expressing recombinant Bg-3βHSD2 together with the indicated substrate and cofactor. Compound numbers correspond to those assigned in Table 1 and are shown in the reaction schemes adjacent the corresponding chromatograms. Products highlighted in blue represent putative metabolites inferred from the established catalytic mechanism of 3βHSD enzymes.

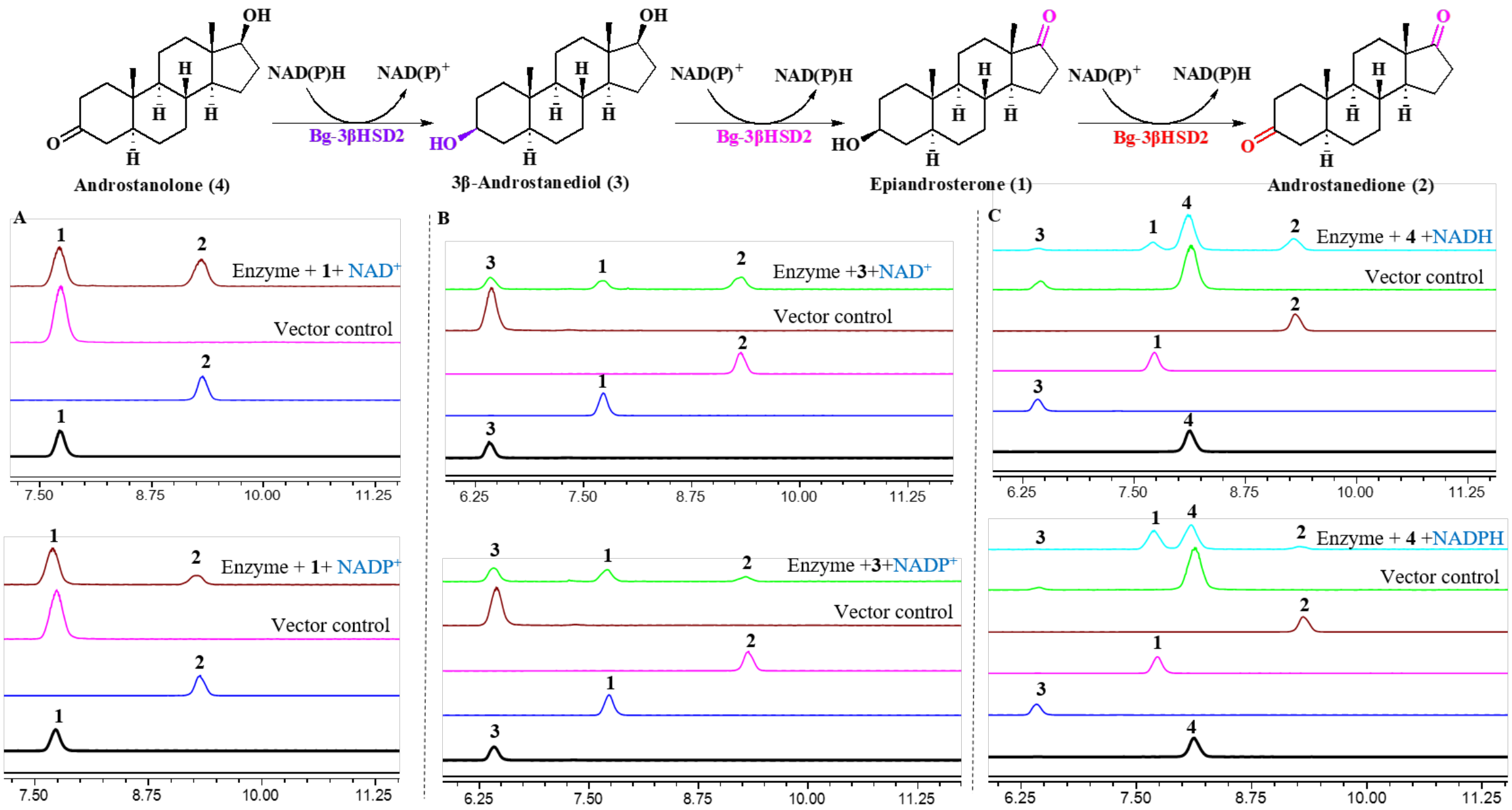

Fig. S11 Representative HPLC chromatograms showing the redox activity of recombinant Bg-3βHSD2 enzyme toward hormone substrates.

Redox conversion of epiandrosterone (**1**) (A), 3β-androstanediol (**3**) (B), and androstanolone (**4**) (C) catalyzed by recombinant Bg-3βHSD2 enzyme, detected by CAD. Oxidation reactions were performed using NAD^+ or $NADP^+$ as cofactors, whereas reduction reactions were performed using NADH or NADPH. In each chromatogram, traces are arranged from bottom to top as follows: authentic substrate standard, authentic product standard, reaction mixture containing crude extracts from *E. coli* cells harboring the empty pCold vector (Vector control), and reaction mixture containing crude extracts from *E. coli* cells expressing recombinant Bg-3βHSD2 together with the indicated substrate and cofactor. Compound numbers correspond to those assigned in Table 1 and are shown in the reaction scheme above the chromatograms.

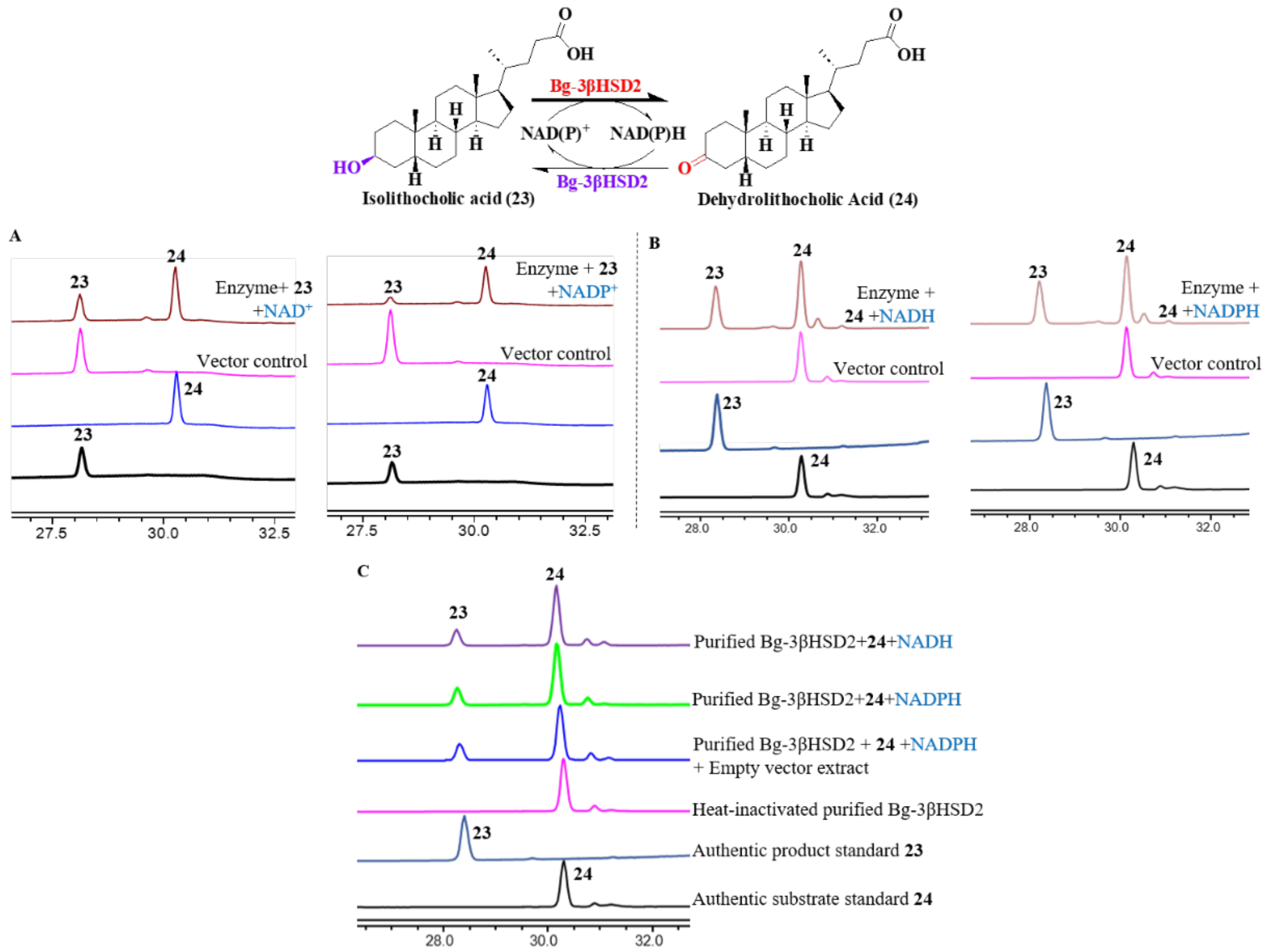

Fig. S12 Representative HPLC chromatograms showing the redox activity of recombinant Bg-3βHSD2 enzyme in both forward and reverse reactions towards bile acid substrate.

Redox interconversion between compound **23** and **24** catalyzed by recombinant Bg-3βHSD2 enzyme, detected by CAD. Oxidation reactions (**23**→**24**) were performed using NAD^+ or $NADP^+$ as cofactors, whereas reduction reactions (**24**→**23**) were performed using NADH or NADPH. In each chromatogram, traces are arranged from bottom to top as follows: authentic substrate standard, authentic product standard, reaction mixture containing crude extracts from *E. coli* cells harboring the empty pCold vector (vector control), and reaction mixture containing crude extracts from *E. coli* cells expressing recombinant Bg-3βHSD2 (A, B) or purified recombinant Bg-3βHSD2 enzyme (C) together with the indicated substrate and cofactor. Compound numbers correspond to those assigned in Table 1 and are shown in the reaction scheme above the chromatograms.

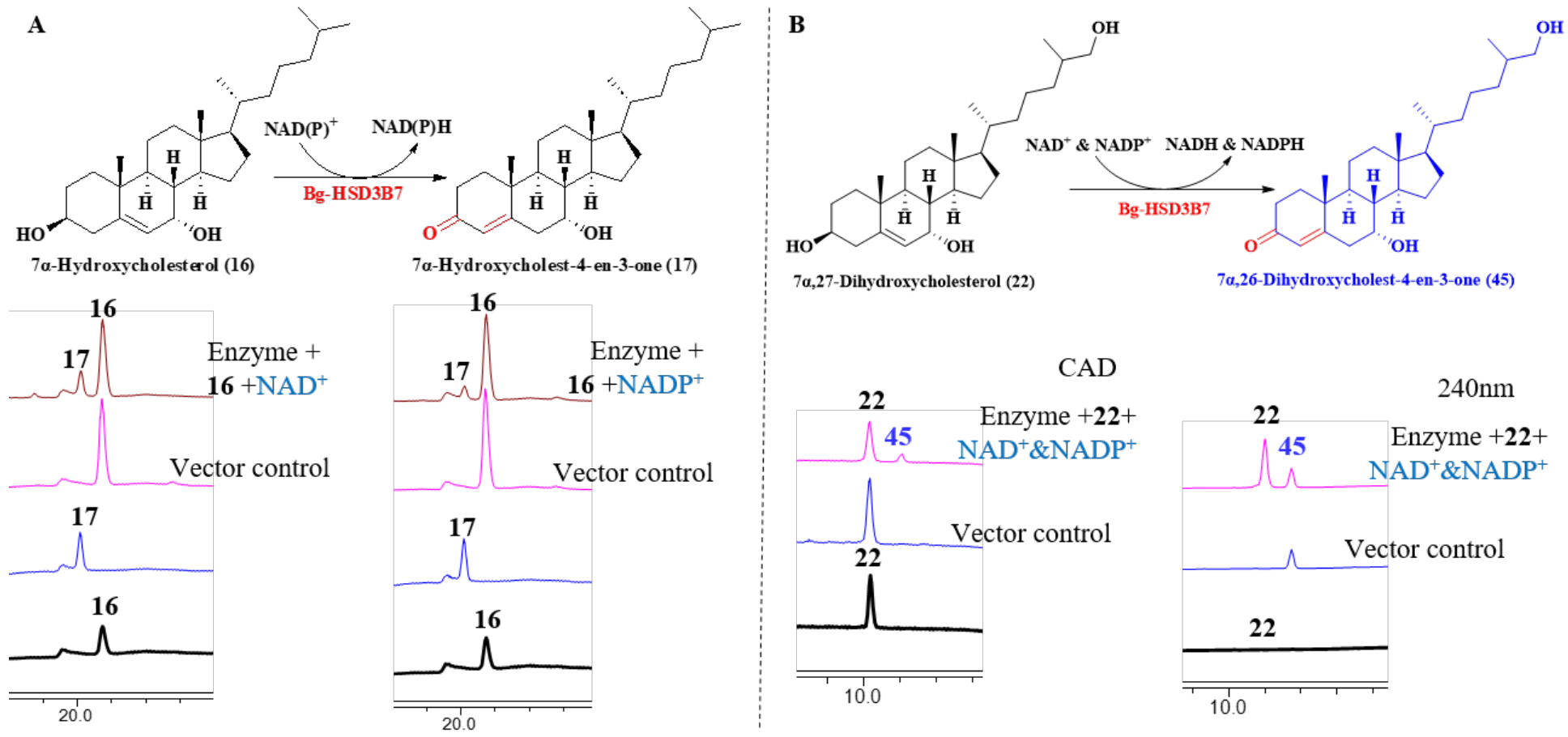

Fig. S13 Representative HPLC chromatograms showing the oxidative activities of Bg-HSD3B7 protein toward sterol substrates.

Oxidative conversion of 7α-hydroxycholesterol (**16**) and 7α,27-dihydroxycholesterol (**22**) catalyzed by recombinant Bg-HSD3B7 protein, with products detected by CAD. In left panel (A), chromatogram traces are arranged from bottom to top as follows: authentic substrate standard, authentic product standard, reaction mixture containing crude protein extracts from *E. coli* carrying the empty expression vector (vector control), and reaction mixture containing crude extracts expressing recombinant Bg-HSD3B7 with the indicated substrate and cofactors. In right panel (B), chromatogram traces are arranged from bottom to top as follows: authentic substrate standard, reaction mixture containing crude protein extracts from *E. coli* cells carrying the empty pCold vector (vector control), and reaction mixture containing crude extracts from *E. coli* cells expressing recombinant Bg-HSD3B7 with the indicated substrate and cofactors. The compound highlighted in blue represents the predicted product inferred from the proposed 3β-HSD catalytic mechanism. Compound numbers correspond to those assigned in Table 1 and are shown in the reaction scheme above the corresponding chromatograms.

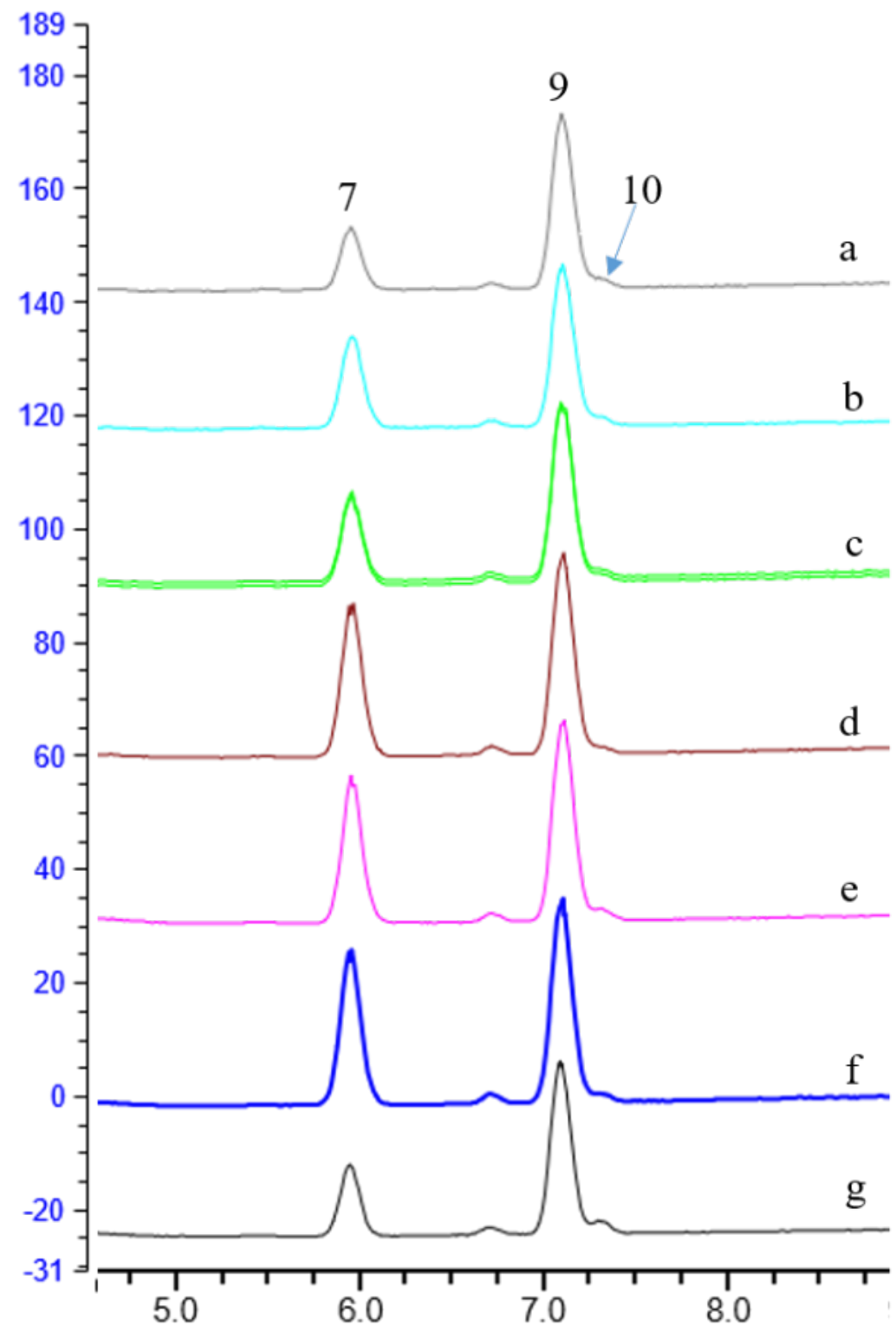

Fig. S14 Representative HPLC chromatograms showing the effects of incubation time, enzyme loading, and cofactor supplementation on the sequential conversion of androstenediol (7) catalyzed by recombinant Bg-3βHSD2 enzyme.

The sequential conversion of androstenediol (**7**) to dehydroepiandrosterone (**9**) and androstenedione (**10**) was analyzed under different reaction conditions, with products detected by CAD. (a) Incubation for 2 h; (b) incubation for 2.5 h; (c) incubation for 3 h; (d) 2 h incubation with 1.5-fold enzyme loading; (e) 2 h incubation with 2-fold enzyme loading; (f) 2 h incubation followed by addition of NAD^+ and further incubation for 0.5 h; (g) 2 h incubation followed by addition of NAD^+ and further incubation for 1 h.
